# Highly efficient catalytic degradation of organic dyes using iron nanoparticles synthesized with *Vernonia Amygdalina* leaf extract

**DOI:** 10.1038/s41598-024-57554-5

**Published:** 2024-03-24

**Authors:** Yohannes Shuka Jara, Tilahun Tumiso Mekiso, Alemayhu Pawulos Washe

**Affiliations:** 1 Department of Chemistry, Natural and Computational Sciences, Madda Walabu University, P. Box 247, Bale Robe, Ethiopia; 2 Department of Chemistry, Natural and Computational Sciences, Hawassa University, P. Box 05, Hawassa, Ethiopia

**Keywords:** Catalytic degradation, Characterization, Iron nanoparticle, Degradation efficiency, Green synthesis, *Vernonia amygdalina*, Methylene blue and Crystal violet dye, Environmental sciences, Natural hazards, Chemistry, Materials science, Nanoscience and technology

## Abstract

Today, nanoscience explores the potential of nanoparticles due to their extraordinary properties compared to bulk materials. The synthesis of metal nanoparticles using plant extracts is a very promising method for environmental remediation, which gets global attention due to pollution-led global warming. In the present study, iron nanoparticles (FeNPs) were successfully synthesized by the green method using *Vernonia amygdalina* plant leaf extract as a natural reducing and capping agent. Biosynthesized FeNPs were characterized with different analytical techniques such as UV–visible, FT-IR, XRD, and SEM. The analysis revealed the formation of amorphous FeNPs with an irregular morphology and non-uniform distribution in size and shape. The average particle size was approximately 2.31 µm. According to the catalytic degradation investigation, the FeNPs produced via the green approach are highly effective in breaking down both CV and MB into non-toxic products, with a maximum degradation efficiency of 97.47% and 94.22%, respectively, when the right conditions are met. The kinetics study exhibited a high correlation coefficient close to unity (0.999) and (0.995) for the degradation of MB and CV, respectively, for the zero-order pseudo-kinetics model, which describes the model as highly suitable for the degradation of both dyes by FeNPs compared to other models. The reusability and stability of biosynthesized nano-catalysts were studied and successfully used as efficient catalysts with a slight decrease in the degradation rate more than four times. The results from this study illustrate that green synthesized FeNPs offer a cost-effective, environmentally friendly, and efficient means for the catalytic degradation of organic dyes.

## Introduction

In science and technology, nanotechnology has emerged as a rapidly emerging concept in recent years and has undergone remarkable development. It is a branch of science and engineering that deals with materials having dimensions in the range of 100 nm or less^1,2^**.** Due to their large surface area-to-volume ratio, nanomaterials are nanoscale in size and are very small particles with improved thermal conductivity, catalytic reactivity, nonlinear optical performance, and chemical stability^3^.

In recent years, many chemical and physical methods have been developed for the synthesis of metallic nanoparticles; these methods serve as important methods in the development of nontoxic, clean, and eco-friendly procedures^4^ and can control, appreciate, and manipulate matter at the level of individual atoms and molecules^5^. The small dimensions of nanoparticles provide extraordinary surface-to-volume ratios, which permit the confinement of electron motion inside boundaries, which is associated with improving their optical properties. This makes them particularly desirable in various application areas, such as medicine^6^, drug delivery^7^, water purification^8^, agriculture^9^, food^10^, solar cells^11^, cosmetics^12^, and textiles^13^.

Metals and metal oxide nanomaterials exhibit vital physicochemical properties, which include increased conductivity, catalytic activity, unusual optical properties, and pyromechanical properties. They also have antimicrobial activity against pathogenic microorganisms^10–13^**.** Nanotechnology is a powerful tool for preventing environmental pollution from different industrial wastes. Due to the increasing use of organic compounds in various industrial and agricultural sectors, environmental safety has become a serious problem for society^14^.

Organic pollutants have considerable adverse effects on aqueous environments. Among the different organic pollutants, dyes are the most frequently used pollutants and are discharged into aqueous environments^15^. Dyes are hazardous, carcinogenic, toxic, and adversely affect human health, the environment, and aquatic ecosystems. They are classified by their structural basis as basic dyes, acidic dyes, azo dyes, disperse dyes, anthraquinone-based dyes, and metal complex dyes^16–18^.

The paint and varnishes, textiles, plastics, ink, cosmetics, and pharmaceutical industries use a large number of organic dyes and their intermediates. Some of these color dyes are extremely toxic and pose a major hazard to the surrounding ecosystem^16,19^. The dye manufacturing industry represents a relatively small part of the overall chemical industry. The worldwide production of dyes is nearly 800,000 tons per year. Approximately 15% of synthetic dyes are lost during different processes in the textile industry^20^. Synthetic dyes are valuable in numerous industries, such as textiles, paper printing, food, pharmaceuticals, leather, and cosmetics. There are more than 10,000 dyes used in textile manufacturing alone, nearly 70% of which are azo dyes; these dyes are complex in structure and are synthetic in nature^21^^**.**^

Aromatic amines such as crystal violet (CV) and methylene blue (MB) can be mobilized by water or sweat, which encourages their absorption through the skin and other exposed areas, such as the mouth^22^. They are used in the textile and paper dye industries, as well as in navy blue and black ink for printing, ballpoint pens, and inkjet printers. It is also used to colorize diverse products, such as fertilizers, antifreeze agents, detergents, and leather^24^. CV and MB have been classified as recalcitrant dyes extensively used in textile processing and as biological stains in veterinary and human medicine. The toxicity of these dyes to human beings and terrestrial and aquatic life causes harmful effects such as cyanosis, vomiting, an increase in heartbeat, quadriplegia, shock, jaundice, and tissue necrosis, prevents the penetration of sunlight into water, reduces photosynthetic function in plants, and harms both aquatic ecosystems and marine vegetation^23–25^.

Synthetic organic dyes and pigments discharged from various industries are responsible for causing substantial environmental pollution^26,27^, as they cannot be degraded by conventional water treatment processes due to their complex aromatic structures, hydrophilic nature, and high stability against light, temperature, water, and chemicals^16,28^. In this view, the degradation of organic dye molecules in waste has gained paramount attention^19^. To date, for the treatment of industrial effluents, various physical and chemical processes, such as ion exchange, adsorption, flocculation, UV radiation, electrochemical reduction, and ionization, have been developed for the elimination of dyes^21,29–31^.

However, most of these processes are associated with secondary pollution problems^23^, complicated procedures, high costs, a pricey setup, relentless energy input^32^, low dye removal efficiency, and the discharge of massive amounts of sludge and toxic intermediates to the environment; additionally, these processes lack practical utility^26,27^. Although photocatalytic oxidative degradation of organic dyes and pollutants is effective, these processes are also slow and inherently energy-consuming. Hence, the development of an effective and eco-friendly protocol for the treatment of industrial effluents is needed. Therefore, designing and synthesizing a catalytic material possessing suitable redox potential between the electron donor and acceptor species to act as an efficient electron relay system is challenging. Interestingly, metal nanoparticles are viable enough to reduce the potential difference because of their high Fermi potentials, which can result in excellent dye degradation efficiency^33,34^. The catalytic reductive degradation of organic dye molecules by metal nanoparticles in the presence of NaBH4 occurs by an electron transfer process. Metal nanoparticles could make the process kinetically feasible by lowering the activation energy^33,35^.

In these processes, there may be a large redox potential difference between the electron donor (BH_4_^−^ ion) and acceptor (dye) species, which can hamper the relay of electrons^27^. In contrast, catalytic reductive degradation of organic dye molecules by metal nanoparticles in the presence of NaBH_4_ is a relatively fast process and was recently used extensively because of its several advantages over other technologies^36,37^**.** This advantage includes the complete degradation of pollutants, simple and easy treatment procedures, and no secondary pollutant generation^38^. For the catalytic degradation of these organic dyes, different nanomaterials, including metal and metal oxide nanoparticles, need to be prepared. Various methods, such as physical, chemical, and biological methods, are used for the synthesis of nanoparticles. The biological method (green method) is the preferred technique because it is more cost-effective, eco-friendly, and easy to use than the other methods^39^. Moreover, plant extracts naturally act as reducing, stabilizing, and capping agents for the synthesis of NPs. Iron nanoparticles (FeNPs) are considered efficient catalysts for dye degradation in the presence of BH_4_^-^ as an ion donor due to their large surface area, high reactivity, and outstanding photochemical stability^33^. The specific surface area of FeNPs is 33.5 m^2^ g^−1^, while that of granular iron is only 0.9 m^2^ g^−1^, which is much less than that of nanosized iron^40^.

*Vernonia amygdalina* is a tree that belongs to the *Asteraceae* family and is a very common plant that grows predominantly in the eastern and western parts of tropical Africa. In addition, it is popularly called bitter leaf in English. *Vernonia amygdalina* grows in most parts of Ethiopia and is known as the ‘grawa’ in Amharic^16^. The leaves of *Vernonia amygdalina* have been found to be relevant in traditional folk medicine as an anthelming agent, a laxative herb, and an antimalarial agent^39^, as they are known as quinine substitutes^40,41^.

Vernonia amygdalina is a plant that has been traditionally used for its antibacterial properties and for maintaining the health of organs such as the kidney and liver, thanks to its therapeutic elements such as saponins, venomygdin, and vernodalin^38^. Leaf decoctions are used to treat fever, malaria, diarrhea, dysentery, hepatitis, and cough; as laxatives; and as fertility inducers^16^. They are also used as medicines for scabies, headaches, and stomachaches. Root extracts are also used as treatments for malaria and gastrointestinal disorders^41^. However, despite its excellent medicinal properties, its pharmacological and environmental actions have not been fully explored. Our research aimed to address this gap by using *Vernonia* *amygdalina* extracts as stabilizers to control the size of FeNPs during synthesis. We hope that this will promote the use of *Vernonia amygdalina* in environmental applications. Our study describes the environmentally friendly biosynthesis and characterization of iron nanoparticles using *Vernonia amygdalina* extracts as reducing and capping agents. Additionally, we report the characterization and application of these nanoparticles in the catalytic degradation of crystal violet and methylene blue.

## Results and discussion

### Visual inspection for the synthesis of iron nanoparticles

**Figure 1:**
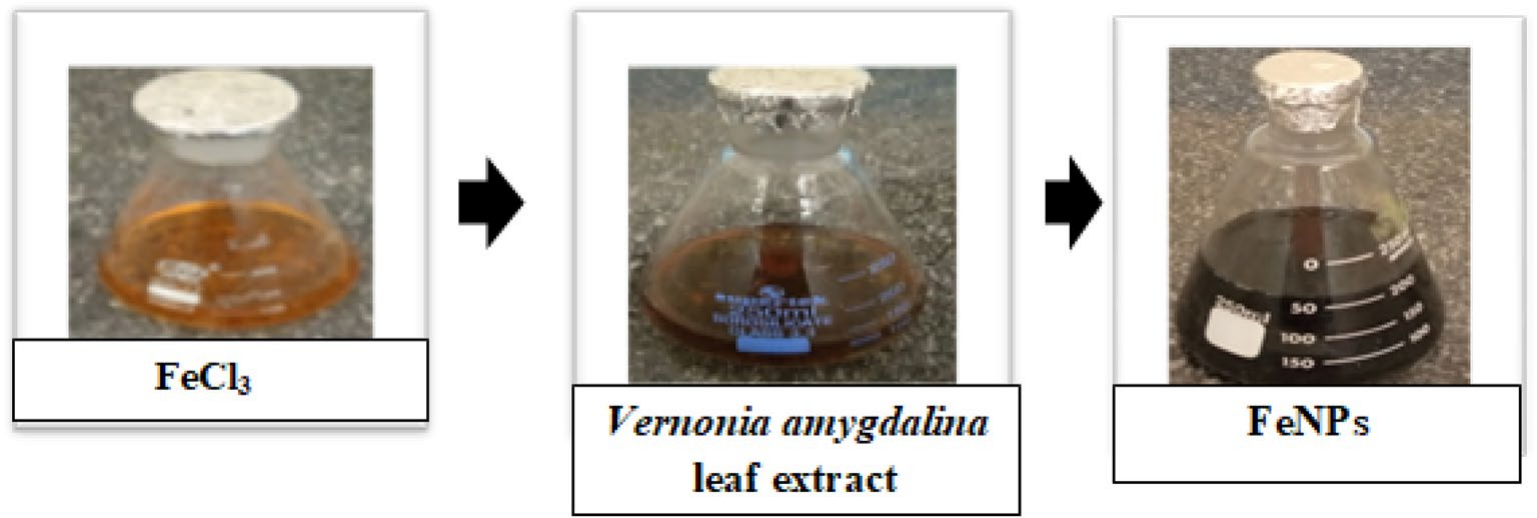
Visual observation of the green synthesis of FeNPs.

NPs exhibit a different array of colors during synthesis. Plant extracts contain several phytochemicals that react with metal ions and convert them to nanoparticles^42^. During the synthesis of the iron nanoparticles, a change in color from light brown to grayish black was observed within 5 min after the addition of *the Vernonia amygdalina* leaf extract, indicating the formation of iron nanoparticles (Fig. 1). After 24 h of reaction, the color change stopped, and precipitation was observed, confirming that the nanoparticle synthesis process was complete^43^.

### Characterization of synthesized nanoparticles

#### Ultraviolet‒visible spectroscopy analysis of FeNPs

**Figure 2:**
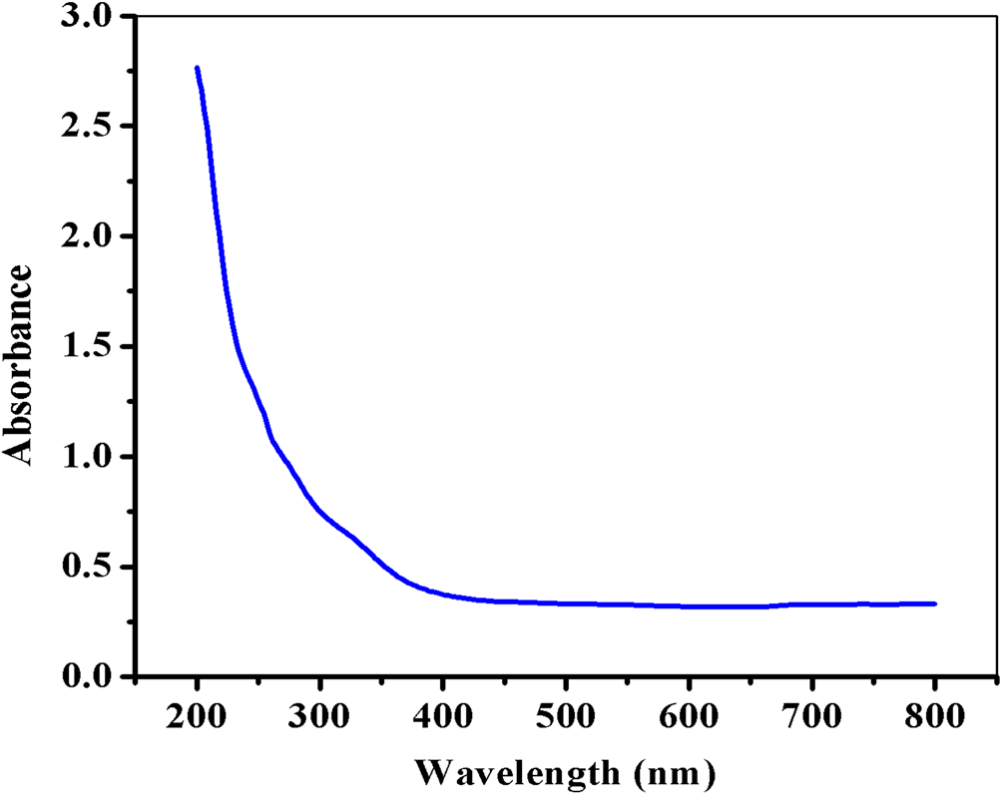
UV‒Vis spectrum of the synthesized FeNPs.

The successful formation of the biosynthesized FeNPs was confirmed by measuring their absorbance at wavelengths ranging from 200 TO 800 nm via UV‒vis spectroscopy. This technique is a powerful tool for detecting the formation of nanoparticles on the surface plasmon resonance (SPR) peak and for calculating their band gap energy. The continuous absorption of the UV‒Vis spectrum in the SPR region confirmed the formation of greenly synthesized FeNPs, which were observed to be nanoscale particles (as depicted in Fig. 2). This observation suggested that the biosynthesized FeNPs may be amorphous and polydispersed. Additionally, the absorbance spectrum of FeNPs obtained using aqueous *Vernonia amygdalina* leaf extract was similar to that of previous reports on FeNPs synthesized using aqueous tea and sorghum extracts^44^, *Catharanthus roseus* leaf extracts^45^, and *Calotropis gigantea* flower extract^46^.

#### Fourier transform infrared (FT-IR) spectral analysis of the FeNPs

**Figure 3:**
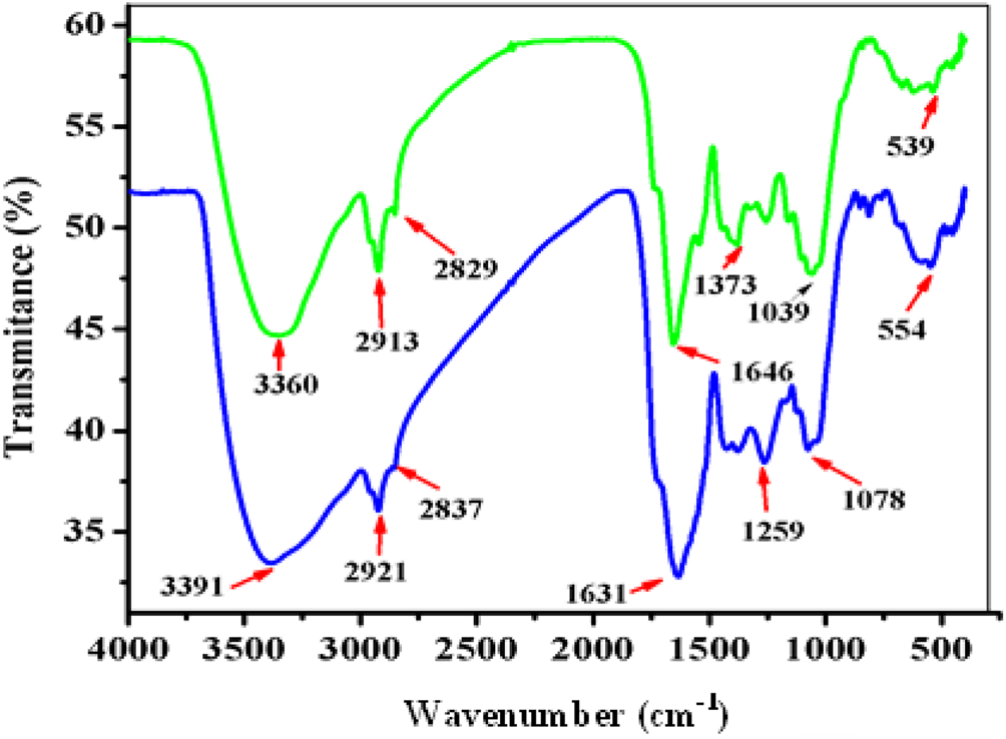
FTIR spectra of the green-synthesized FeNPs (blue spectrum) and the *Vernonia amygdalina* leaf extract (green spectrum).

Fourier transform infrared (FT-IR) spectroscopy was employed in this study to determine the surface composition of the synthesized FeNPs, mainly the specific functional groups that may contribute to the formation of FeNPs. Thus, this technique provides information on the interactions between phytochemicals present in plant extracts and the metal ions responsible for the production and capping of iron nanoparticles. This was confirmed in terms of the individual band intensities observed in the extracts and their corresponding nanoparticles. The FT-IR spectra of the *Vernonia amygdalina* leaf extract and the synthesized FeNPs are shown in Fig. 3. The FT-IR spectrum of the leaf extract showed intense bands at 3360.35 cm^−1^ (O–H stretching vibrations), 2913.26 cm^−1^ (C–H and CH_2_ vibrations of aliphatic hydrocarbons), 2829.26 cm^−1^ (C–H stretching of aldehydes), 1646.95 cm^−1^ (C = C stretching vibrations), 1373.11 cm^−1^ (C–O stretching of the ester group), and 1039.88 cm^−1^ (due to C–O bonds). The FT-IR spectrum of FeNPs displays intense bands at 3391.35 cm^−1^ (O–H stretching vibrations), 2921.06 cm^−1^ (C–H and CH_2_ vibrations of aliphatic hydrocarbons), 1631.74 cm^−1^ (C = C stretching vibrations), 1259.83 cm^−1^ (C–O stretching of the ester group) and 1078.27 cm^−1^ (due to C–O bond stretching of the primary alcohol), as well as absorption bands at approximately 554 cm^−1^, which corresponds to the Fe–O bond. A comparison of the FT-IR spectra of *Vernonia amygdalina* and the Fe NPs revealed a band shift toward higher frequencies from 3360.96 to 3391.06 cm^−1^ (O–H stretching vibrations), 2913.26 to 2921.06 cm^−1^ (C–H and CH_2_ vibration of aliphatic hydrocarbons) and 2829.88 to 2837.87 cm^−1^ (C–H starch of aldehyde), suggesting the possible association of the polyphenol compounds in the leaf extract with metal ions in the formation of FeNPs. The absorption peak of the nanoparticles was similar to that of the extracts, and the differences in intensities suggested the importance of functional groups in the reduction and capping processes. The soluble elements present in the *Vernonia amygdalina* leaf extract could have acted as capping agents preventing the aggregation of FeNPs^47^.

#### X-ray diffraction (XRD) analysis of the FeNPs

**Figure 4:**
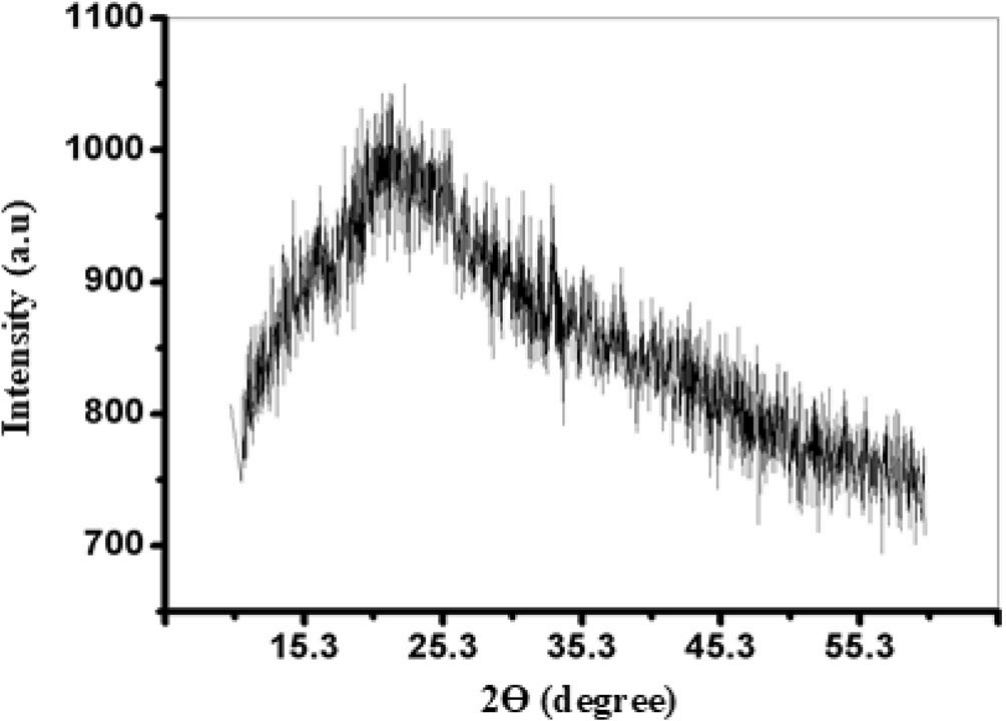
X-ray diffraction (XRD) pattern for the FeNPs.

X-ray diffraction (XRD) analysis was employed to explore the surface crystallinity of the biosynthesized iron nanoparticles, revealing predominantly amorphous FeNPs supported by indistinct diffraction peaks (Fig. 4). Similarly, prior investigations have reported the production of amorphous FeNPs using leaf extracts from diverse plant sources, such as eucalyptus, pomegranate, and cherry^48^. The presence of organic components, derived from the leaf extract, is indicated by a broad shoulder peak observed within the 15° to 30° range of 2θ values, suggesting their role as capping and stabilizing agents for the synthesized iron nanoparticles^49^. The XRD spectrum of FeNPs displays broadening diffraction and a lack of well-defined peaks, signifying the polydispersion of the nanoparticles, and the presence of multiple peaks at different angles suggests a variation in size. This pattern aligns well with the findings from the SEM images and PSA analysis. Notably, analogous FeNP patterns were observed in studies harnessing eucalyptus, pomegranate, and cherry aqueous extracts^50,51^.

#### SEM and PSA for biosynthesized FeNPs

**Figure 5:**
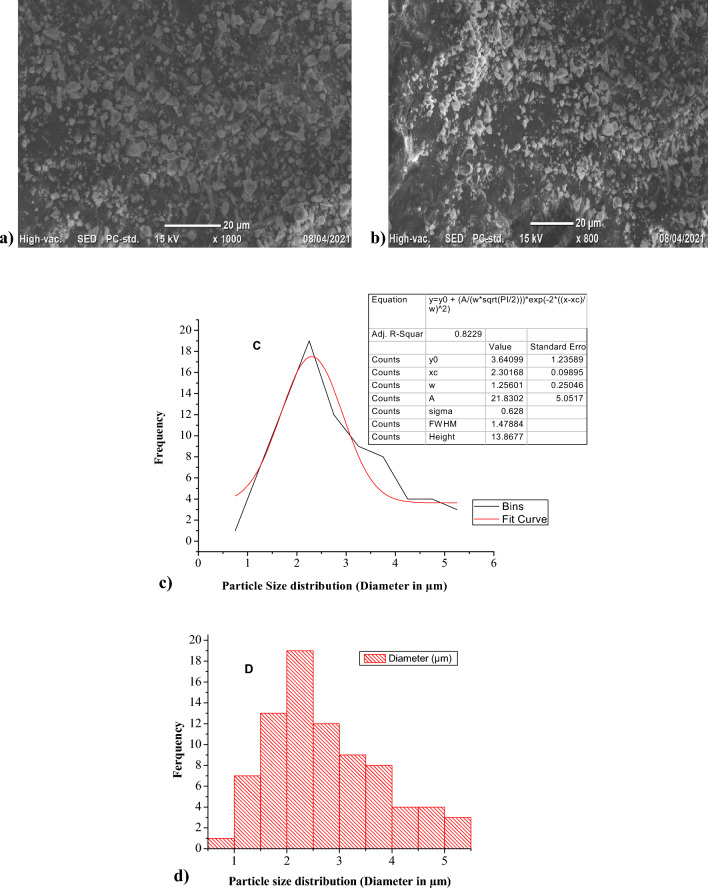
SEM images (**a** and **b**); Histogram of PSA (**c** and **d**) in the biosynthesized FeNPs.

The morphology, microstructure, and particle size distribution of the synthesized FeNPs were assessed using scanning electron microscopy (SEM) images and particle size analysis (PSA). SEM images of FeNPs synthesized with *Vernonia amygdalina* leaf extract at various magnifications are shown in Fig. 5a and b, revealing the successful synthesis of FeNPs. According to these images, the formed nanoparticles exhibit an irregular morphology and lack a uniform distribution in shape and size. By utilizing Image J software through SEM, the average particle size distribution of the FeNPs was estimated to be approximately 2.31 µm, as displayed in Fig. 5c and d. The analysis indicated that approximately 90% of the particles fell within the 1–4 µm range, while the remaining particles were outside this range. This result is consistent with that of a previous study on the biosynthesis of FeNPs from loquat *Eriobotrya japonica* leaves, which reported an average particle diameter of 114.3 nm and 0.114.3 µm^52^^**.**^

The biosynthesized FeNPs from the leaf extracts of *Vernonia amygdalina* were found to be polydispersed based on the XRD and UV‒Vis spectra, with a majority of them exhibiting a variable size with a smaller diameter. The results were confirmed by identifying size variation, with an average diameter of approximately 2.31 µm for the biosynthesized FeNPs, suggesting their relatively smaller size.

### Evaluation of the catalytic activity of FeNPs for CV and MB dye degradation

#### Control experiment

**Figure 6:**
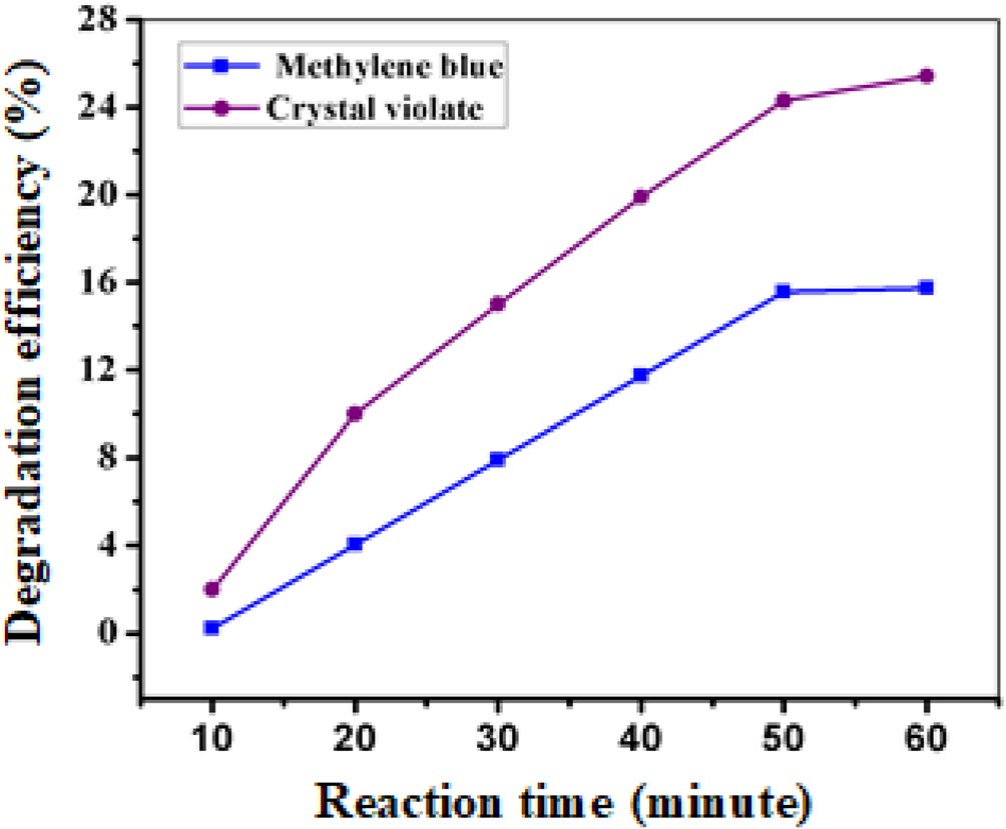
Degradation of CV and MB in the absence of 10 ppm initial FeNP concentration.

Sodium borohydride is known to facilitate the reduction of crystal violet and methylene blue. However, when used in the absence of FeNPs, this process occurs slowly. As depicted in Fig. 6, after 60 min, only 25.10% and 15.73% of the crystal violet and methylene blue, respectively, were reduced. This is due to the large difference in redox potential between the donor and the acceptor, which makes the reduction thermodynamically favorable but not kinetically favorable. The addition of FeNPs as a nanocatalyst provides an alternative route with low activation energy for the reduction reaction, reducing the kinetic barrier and making the reaction kinetically favorable. Furthermore, the FeNPs provide a suitable surface for binding crystal violet and methylene blue particles and borohydride ions (BH_4_^−1^) to interact with each other, leading to the formation of decomposition products. These nanoparticles make the degradation of crystal violet and methylene blue kinetically feasible, resulting in complete reduction within a short period of time^53,54^.

#### Effect of catalyst loading

**Figure 7:**
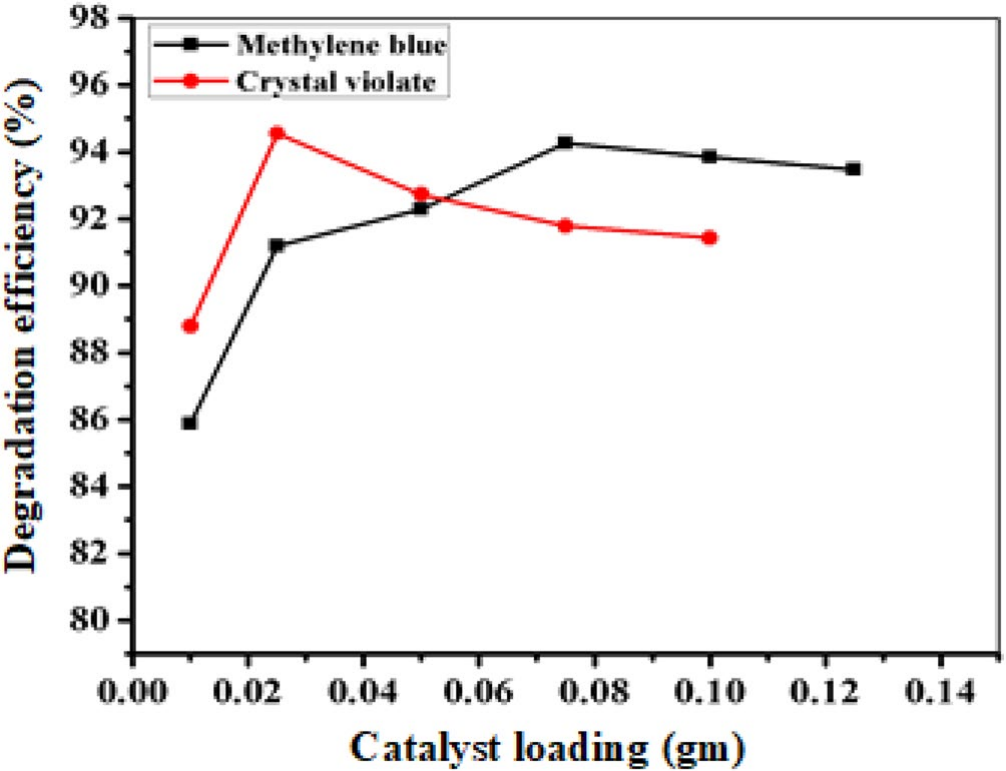
Effect of catalyst loading on the degradation of CV and MB.

To determine the optimal quantity of catalyst required for the efficient degradation of crystal violet and methylene blue, various concentrations of aqueous solutions containing the dyes at a concentration of 10 ppm were tested in the presence of varying amounts of the FeNP catalyst. The results, which are presented in Fig. 7 and ‘Tables S1 and S2’, indicated that the degradation efficiency increased as the amount of FeNP catalyst increased from 0.010 to 0.025 gm for crystal violet and from 0.010 to 0.075 gm for methylene blue. This can be attributed to the greater availability of surface active sites for BH_4_^−1^ ion and dye molecule adsorption with increasing FeNP dose^37^. Based on these findings, optimum catalyst loads of 0.025 gm for crystal violet and 0.075 g for methylene blue were identified, resulting in degradation percentages of 95.7% and 93.15%, respectively. It is worth noting that beyond the optimum catalyst load, the efficiency decreases due to the aggregation of nanoparticles at high catalyst doses, which may contribute to a lower degradation percentage by reducing the number of active sites^55^ and decreasing the electron density.

#### Initial concentration of dyes

**Figure 8:**
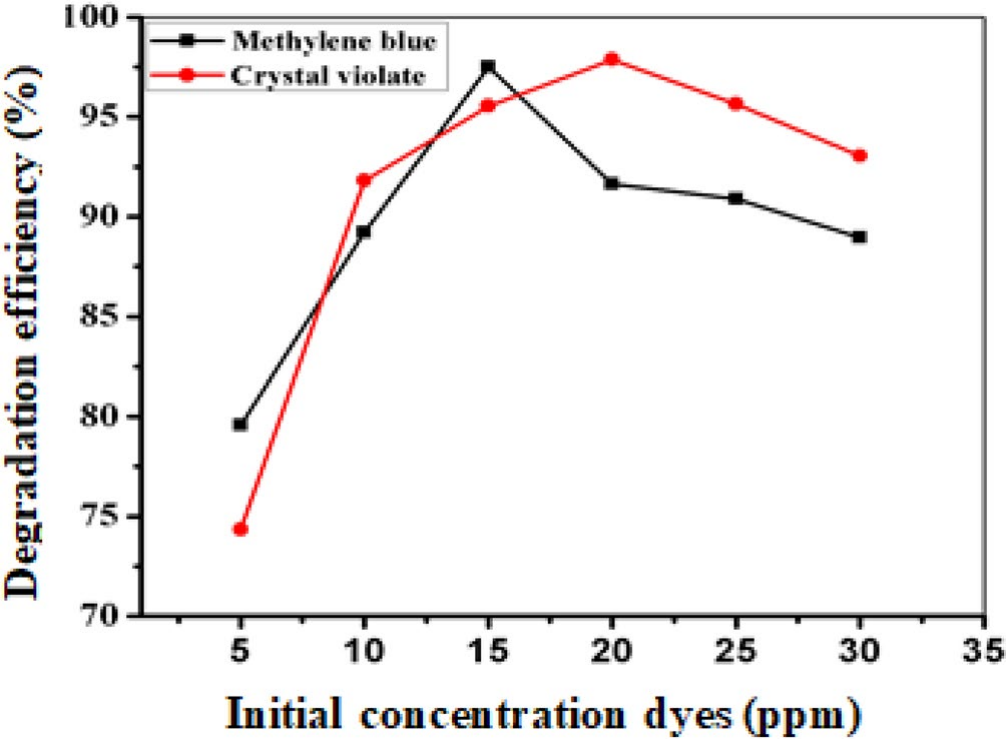
Effect of initial concentration on the catalytic degradation of CV and MB.

The results of the present study, which involved examining six different concentrations of aqueous solutions containing crystal violet and methylene blue ranging from 5 to 30 ppm, are presented in Fig. 8 and Tables S3 and S4. The findings indicate that the degradation efficiency increased to an optimum value with increasing dye concentration from 5 to 20 ppm for crystal violet (97%) and from 5 to 15 ppm for methylene blue (93%). However, the rate of degradation decreased with a further increase in dye concentration, as this inhibited the electron transfer process on the nanoparticle surface between NaBH_4_ and the dye, thereby slowing down and reducing the number of active sites on the catalyst^20^.

#### Effect of NaBH_4_ concentration

**Figure 9:**
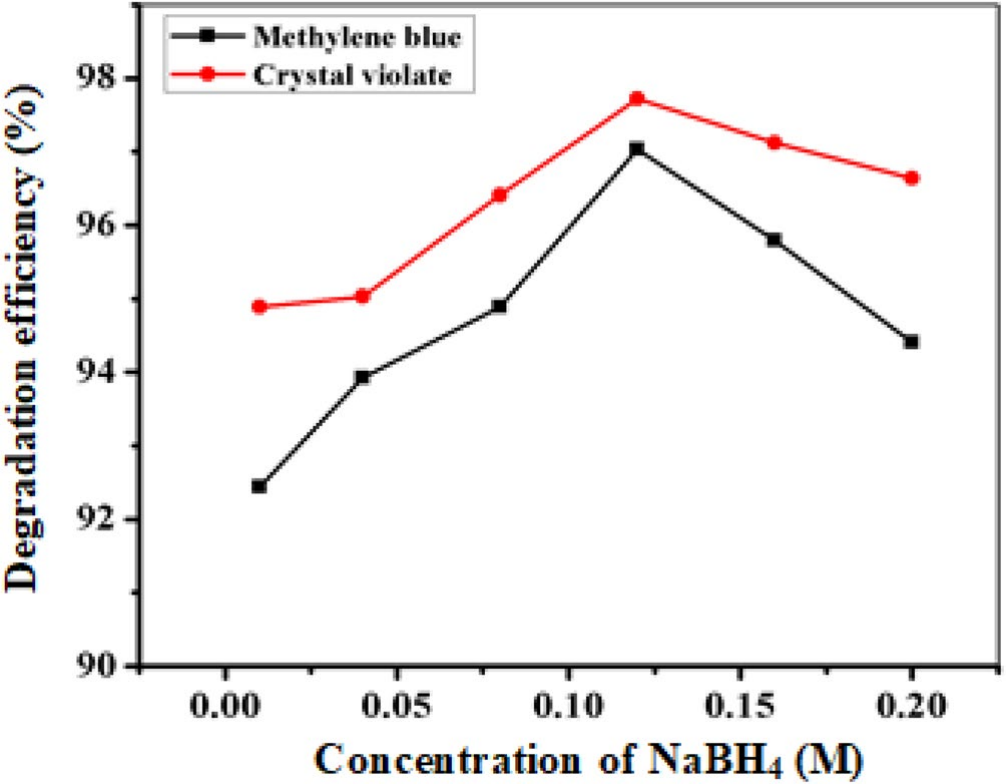
Effect of sodium borohydride concentration on the catalytic degradation of CV and MB.

The concentration of NaBH_4_ was varied to determine the optimal concentration for degrading crystal violet and methylene blue while maintaining a fixed nanoparticle dose and dye concentration. The results revealed an increase in degradation with increasing NaBH_4_ concentration from 0.01 to 0.12 M, reaching a maximum efficiency at 0.12 M (Fig. 9, additional ‘Tables S9 and S10’). Hence, 0.12 M was identified as the critical concentration, highlighting the necessity of an appropriate BH_4_^−1^ ion concentration for optimal nanoparticle degradation. Moreover, the higher NaBH_4_ concentration elevated the local electron density on the FeNP surface, potentially leading to accelerated reaction rates^56^.

#### Effect of reaction time

**Figure 10:**
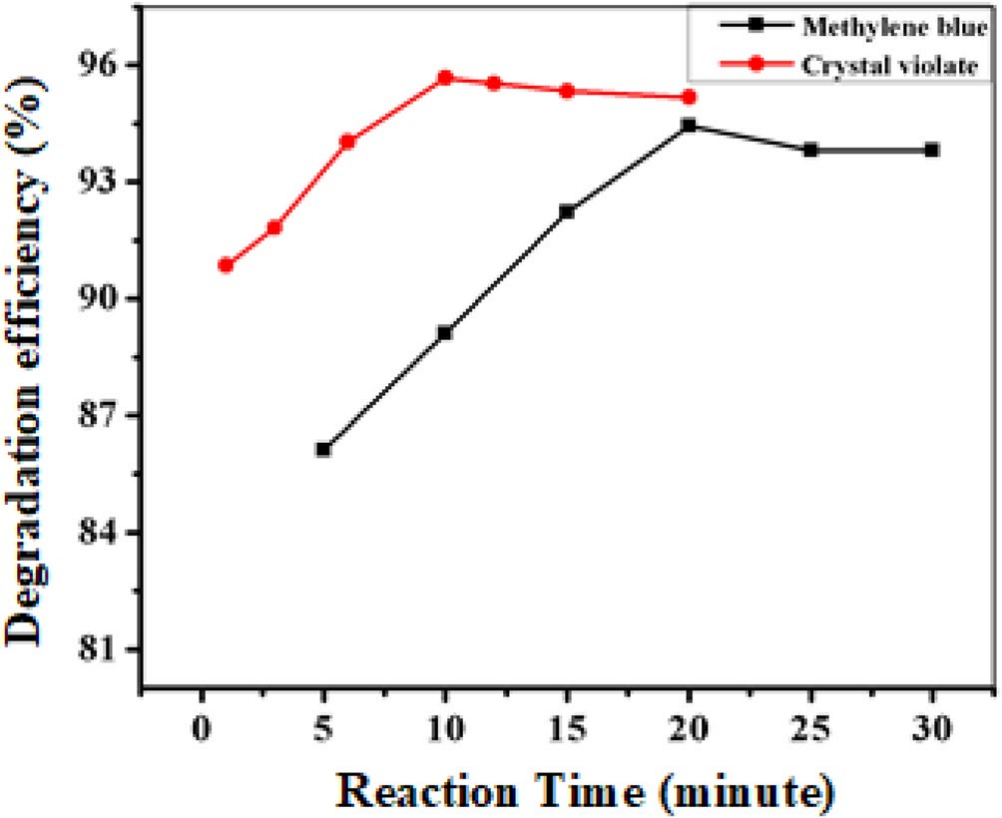
Effect of reaction time on the catalytic degradation of CV and MB.

The response over time (Fig. 10, see Tables S5 and S6) revealed that the degradation efficiency of both dyes increased with reaction time, peaking at 10 min for crystal violet (95.70% degradation) and 15 min for methylene blue (95.15% degradation). Beyond these durations, the efficiency plateau due to the active sites on the photocatalyst surface may become saturated or deactivated over time, suggesting that extended reaction times did not yield further enhancements in degradation efficiency^57^.

#### Effect of pH

**Figure 11:**
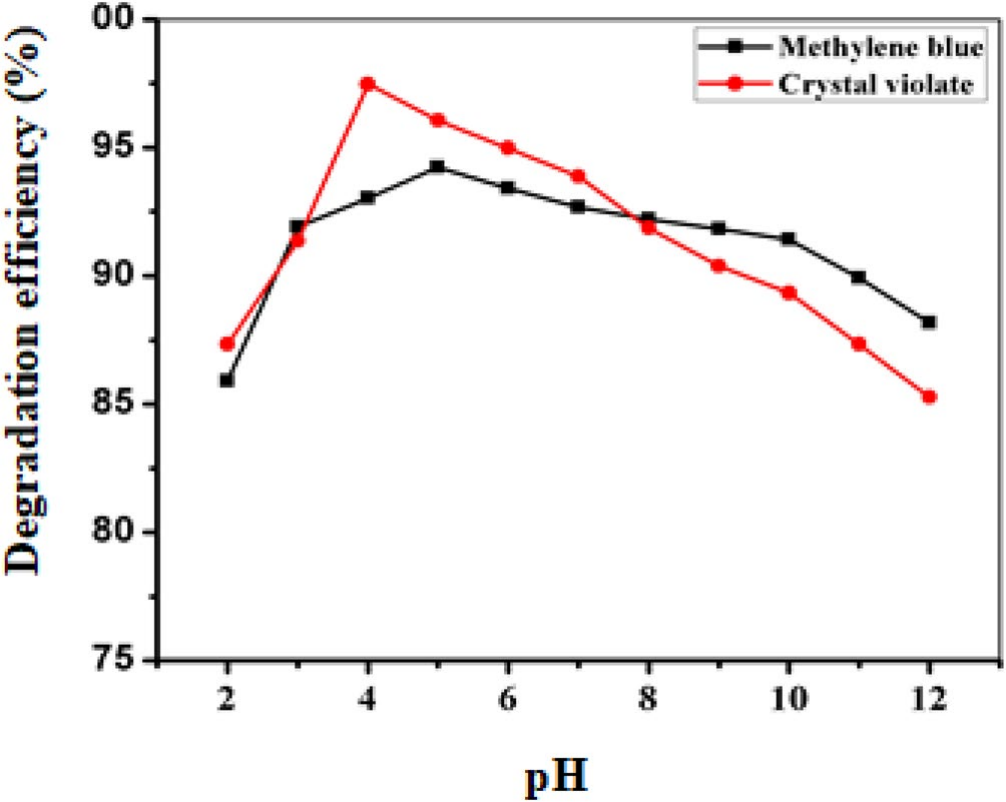
Effect of pH on the catalytic degradation of CV and MB under optimized conditions for all the other parameters.

The pH contributes to determining the acidity or basicity of a solution and is an important parameter affecting catalytic degradation. This study explored the optimization of pH levels within a range from 2 to 12 using initial concentrations of 20 ppm crystal violet, 15 ppm methylene blue, and 0.025 and 0.075 g of FeNPs. The results (Fig. 11, see Tables S7 and S8) revealed that the highest degree of crystal violet degradation (97.47%) occurred at pH 4, while the highest degree of methylene blue degradation (94.22%) occurred at pH 5. Subsequently, increasing the pH to 12 resulted in a gradual decrease in degradation percentages for both dyes (efficiency increases in acidic medium and decreases in basic medium), potentially due to the formation of ferrous hydroxide, which could have occupied active sites on the FeNPs, subsequently reducing their reduction capacity^58^.

### Catalytic reduction and degradation of CV and MB

**Figure 12:**
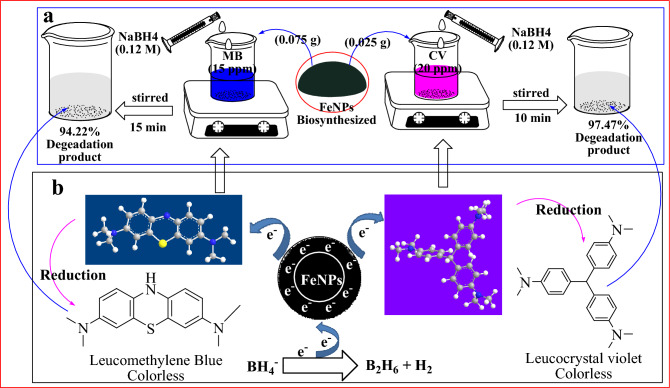
Schematic diagram of the catalytic reduction reaction (**a**) and mechanism of the catalytic degradation of CV and MB by the NaBH_4_ and FeNP samples (**b**).

The reduction of dyes by FeNPs can be explained through the electron transfer effect. Iron, a good conductor, can facilitate the transfer of electrons between donors and receptors. Thus, this catalytic process is mediated by iron nanoparticles via a redox mechanism, allowing for the transfer of electrons from the donor (BH_4_^−^) to the acceptors (CV and MB). As depicted in Fig. 12a, the reduction of dyes in the presence of FeNPs is initiated by NaBH_4_. Subsequently, with the assistance of FeNPs as a catalyst, electrons are transferred from BH_4_^−^ to CV and MB, leading to the reduction of CV and MB into colorless and nontoxic LCV (leucocrystal violet) and LMB (leucomethyene blue), respectively, by removing chromophore groups such as azo, nitro, N–C and S–C conjugated systems, as indicated in Fig. 12b (see F1-SI for the detailed mechanism). The reduction of CV and MB by the FeNP catalyst in the presence of NaBH_4_ can be elucidated using the Langmuir–Hinshelwood model^59^. According to this model, the initially adsorbed BH_4_^−^ ions donate electrons to the FeNPs (as shown in Eq. 1). As a result, a negatively charged layer develops around the FeNPs^60^. The reduction reaction at the FeNP surface is followed by the transfer of electrons from the FeNPs to cationic dye molecules through electrostatic interactions^59^. Previous work on PNP reduction by Ag/SiO_2_ NC catalysts has also shown that these catalysts adhere to the Langmuir–Hinshelwood mechanism^61^. This model involves both molecules adsorbing onto the catalyst surface before engaging in the bimolecular reaction. When NaBH_4_ is in excess, electrons and hydrogen are transferred to the surface of the nanocatalyst, where they control the rate of the reaction through the adsorption of dyes and borohydride ions (BH_4_^−^) on the nanocatalyst surface.

1 $${\text{2BH}}_{{4}}^{ - } + {\text{FeNPs}} \to \left[ {{\text{FeNPs}}} \right]^{{{2} - }} + {\text{B}}_{{2}} {\text{H}}_{{6}} + {\text{H}}_{{2}}$$

The present study investigated the efficacy of an FeNP catalyst in the presence of NaBH_4_ as a reducing agent for the degradation of MB and CV dyes, which are commonly found in industrial effluents. The reduction process was found to be highly efficient, with removal efficiencies of 94.22% for MB and 97.47% for CV within 10 and 20 min, respectively, under optimized experimental conditions (Fig. 11). NaBH_4_ was preferred as the reducing agent due to its excellent electron-donating ability, facilitated by BH_4_^−^ ions.

The cationic nature of the CV and MB dyes facilitated their strong attraction toward FeNPs, which generated a negative layer that facilitated quick electron transfer to the dye molecules. NaBH_4_ also serves as a source of hydrogen, which targets organic dyes after electron transfer to FeNPs. The resulting reaction produced reduced, colorless forms of methyl blue and crystal violet^27^. Subsequently, LCV and LMB desorbed spontaneously from the surface of the FeNPs and diffused into the solution due to weaker electrostatic interactions between the FeNPs and the colorless degradation products^62^.

### Kinetics studies

**Figure 13:**
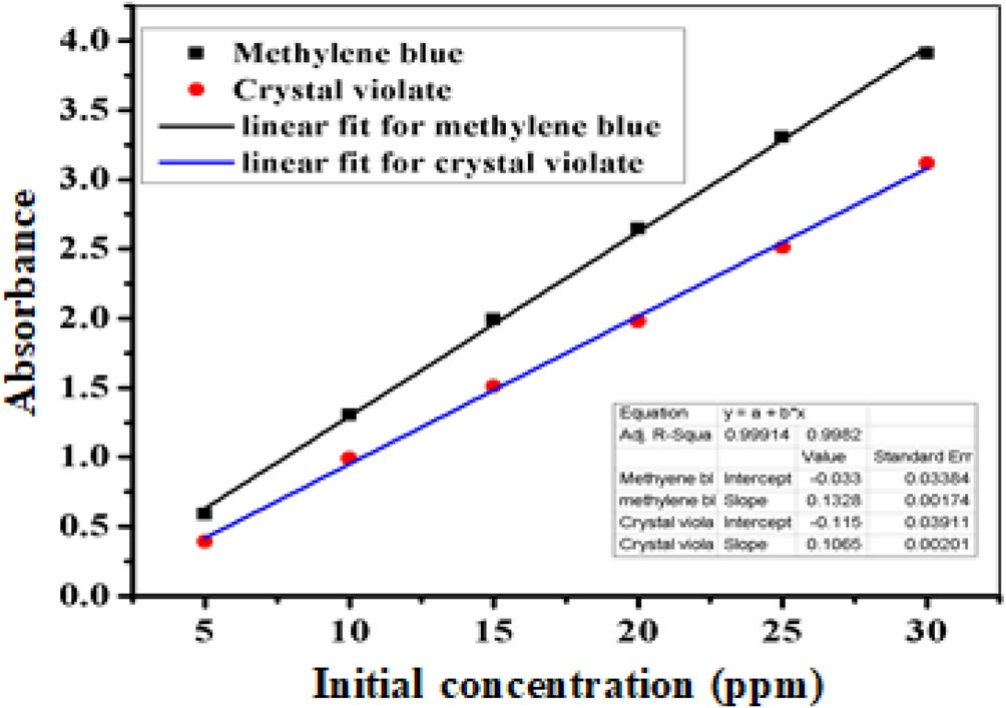
Calibration curves of crystal violet and methylene blue.

In this study, various kinetic models were used to analyze the experimental data and determine the degradation mechanism of dyes. Specifically, three kinetic models were established: the pseudo zero-order, pseudo first-order, and pseudo second-order models. The degradation reactions were conducted under optimal experimental conditions at different time intervals ranging from 5 to 20 min while keeping all the other parameters constant. To investigate the pseudo kinetics, calibration curves were constructed, as depicted in Fig. 13. These curves were used to calculate the concentration of the dyes after degradation at different time intervals. The data for the calibration curves of crystal violet and methyl blue are presented in Table ‘S13’.

Pseudo kinetic experiments were also conducted, and the data used for calculating the residual concentration of the dyes are presented in the table in ‘‘Table S14’’. From Table S14, the final concentrations of both dyes were calculated using their respective calibration curves, and other quantities used for investigating the pseudo kinetics were calculated and are shown in Table 1.

**Table 1:** Data for examining the pseudo kinetics of heterogeneous catalysis by FeNPs.

Dyes	C_o_ (ppm)	Time (min)	${C}_{t}$ (ppm)	${C}_{o}-{C}_{t}$	$\frac{{C}_{o}}{{C}_{t}}$	$ln\frac{{C}_{o}}{{C}_{t}}$	$\frac{1}{C}_{t}-\frac{1}{C}_{o}$
Crystal violet	20	1	2.77	17.23	7.22	1.97	0.31
		3	2.58	17.42	7.75	2.05	0.33
		6	2.20	17.69	9.09	2.20	0.43
		10	1.76	17.97	11.36	2.28	0.49
Methylene blue	15	5	2.33	12.66	6.43	1.86	0.36
		10	1.89	13.11	7.97	2.07	0.46
		15	1.42	13.57	10.56	2.35	0.64
		20	1.09	13.97	13.77	2.62	0.85

#### Pseudo zero-order, pseudo first order, and pseudo second order kinetic models

**Figure 14:**
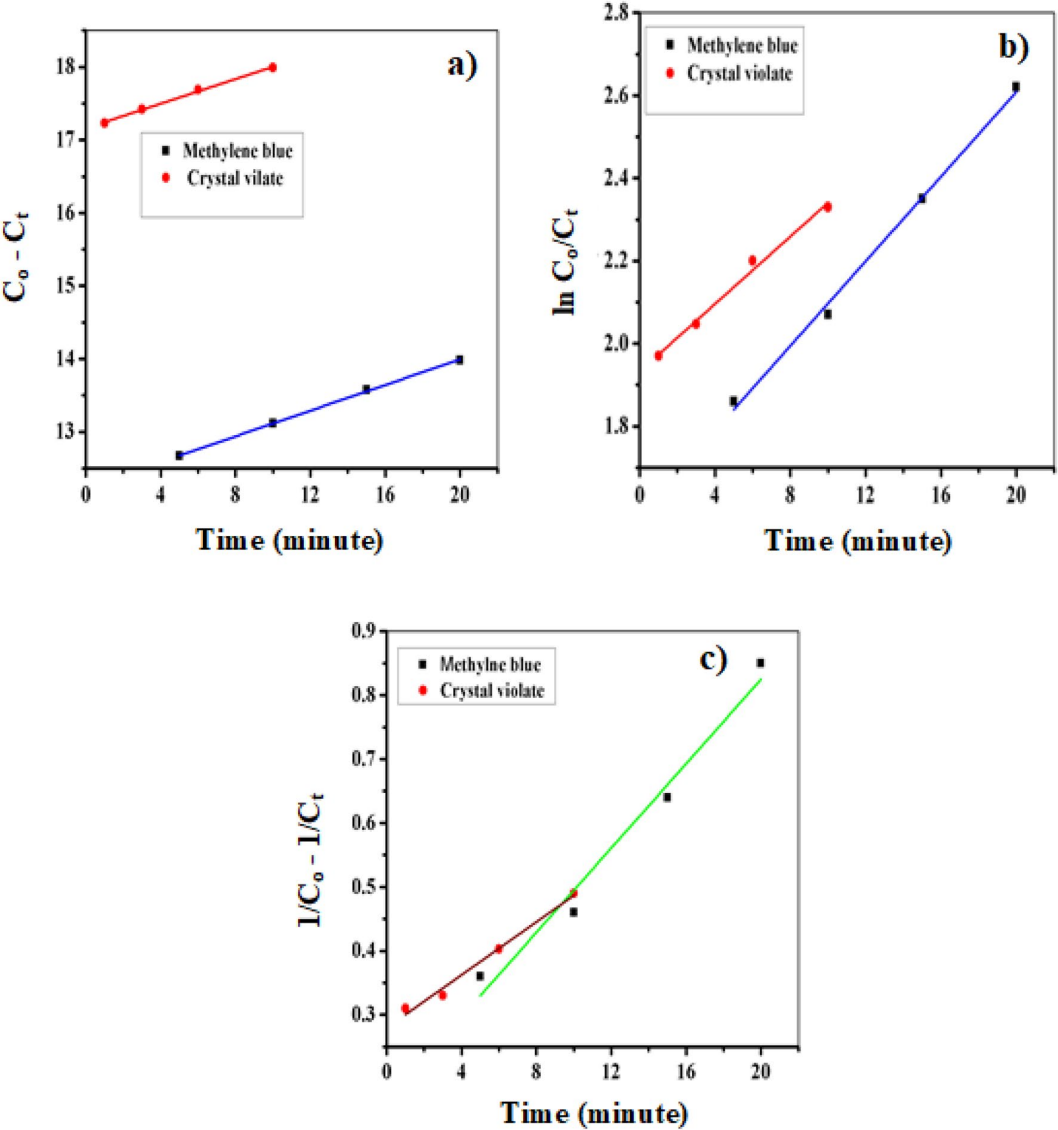
Pseudo zero-order (**a**), pseudo-first-order (**b**) and pseudo-second-order kinetic models for the catalytic degradation of CV and MB by FeNPs with NaBH_4_.

To investigate the degradation kinetics of crystal violet and methylene blue dyes by FeNPs, Eqs. (4), (5), and (6) were linearized and plotted as C_o_ − C_t_ against time (t) and ln [C_o_/C_t_] against time, and the plots 1/C_o_ − 1/C_t_ versus time (t) were generated for the pseudo zero-order, pseudo-first-order, and pseudo-second-order kinetics models, respectively. Linear graphs with correlation coefficients for all the kinetics models are presented in Fig. 14a,b, and c. The correlation coefficients and rate constants for each kinetic model were calculated and are summarized in Table 2. According to the results of the analysis of pseudo zero-order kinetics, compared with the other models, the FeNPs exhibited high correlation coefficients close to unity (R^2^ = 0.999 and R^2^ = 0.995) for the degradation of methylene blue and crystal violet, respectively. These findings suggest that the degradation process best-fitting a pseudo zero-order kinetics model. Specifically, a pseudo zero-order reaction was observed when the surface of the catalyst reached saturation, and the degradation rate became independent of the dye concentration and remained constant over time. The results presented in Table 2 for pseudo-first-order kinetics reveal a relatively high correlation coefficient for only the catalytic degradation of methylene blue. These findings suggest that the catalytic degradation process occurring on the surface of the FeNP catalyst follows pseudo-first-order kinetics, where the rate of catalytic degradation and concentration of dye are directly proportional. Compared to those of other pseudo kinetic models, such as pseudo zero-order and pseudo-first-order models, the correlation coefficient of the pseudo-second-order model (as shown in Fig. 14c) is lower for both dyes. This indicates that the catalytic degradation of both dyes does not fit the pseudo-second-order model. In general, the high correlation coefficient suggested that the zero-order pseudo kinetic model is a suitable model for describing the degradation kinetics of both dyes by FeNPs. This finding is important because it indicates that FeNPs can be used as effective catalysts for the degradation of these dyes in wastewater.

**Table 2:** Rate constant (k) and correlation coefficient (R^2^) calculated for the kinetic model.

Types of model	Catalyst FeNPs			
	Crystal violet		Methylene blue	
	K	R^2^	K	R^2^
Pseudo zero order	0.084	0.995	0.088	0.999
Pseudo first order	0.041	0.988	0.051	0.994
Pseudo second order	0.021	0.981	0.033	0.966

### Study of the reusability of iron nanoparticles

**Figure 15:**
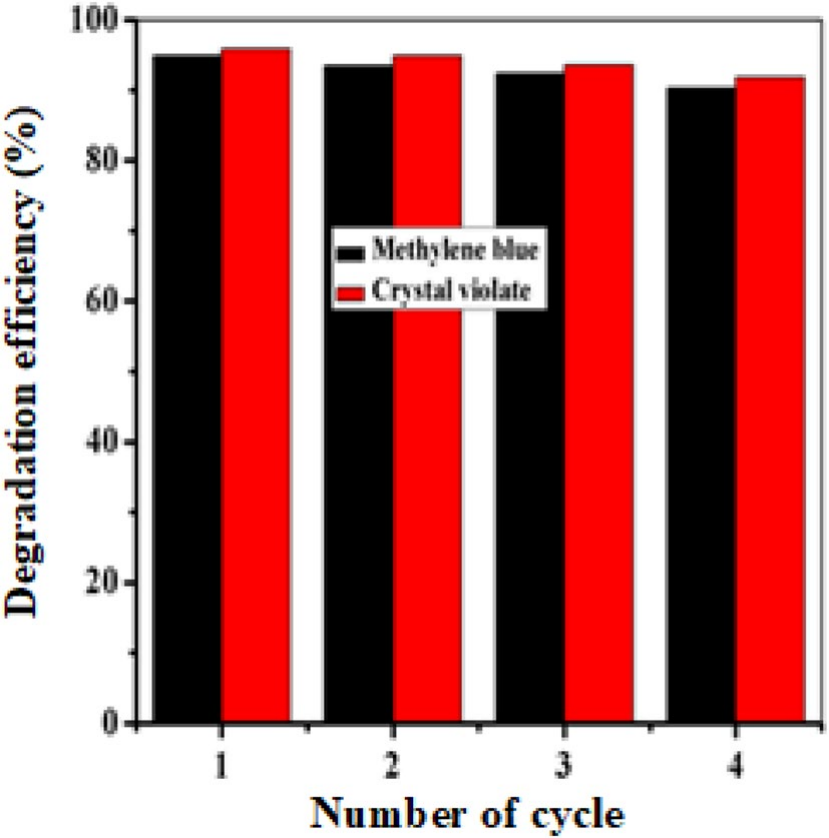
Reusibility of FeNPs.

Currently, the reusability of catalysts is a significant issue because a large number of catalysts are deactivated after the first or second cycle and are subsequently discarded. The reusability and stability of catalysts are vital for determining their performance, as an active and stable catalyst could considerably decrease the cost of the process. Here, crystal violet and methylene blue were used to study the reusability of FeNPs under optimum experimental conditions. The reusability of the FeNPs was tested four times. Figure 15 and ‘‘Tables S11 and S12’’ show that the reusability of the FeNPs was good. The findings of this study indicate that the FeNPs synthesized through green methods were relatively stable and could serve as effective catalysts, with only a slight reduction in their degradation rate after being used four times. These results are consistent with those of a previous study in which amorphous FeNPs synthesized from an aqueous extract of *Boswellia serrata*, a renewable natural resource, were utilized. In that study, the FeNPs were found to retain their catalytic activity even after being recycled up to five times, with only a modest decrease in their effectiveness^63^. Thus, the results of both studies suggest that green synthesized FeNPs have the potential to be used as efficient and sustainable catalysts in various applications.

## Materials and methods

### Chemicals and reagents

The following analytical grade reagents were used directly as obtained without any further treatment throughout the work: ferric chloride, FeCl_3_ (99.99%, Sigma‒Aldrich, Indian) were used for the synthesis of iron nanoparticles as metal ion precursors. Crystal violet (AR, Samir Tech-Chem, Ltd., India) and methylene blue (AR, UniChem Ltd., India) dyes were used for the evaluation of the catalytic activity of the prepared iron and nanoparticles, and sodium borohydride, NaBH_4_ (97% extra pure Merck, India), ethanol, CH_3_OH (97% extra pure Sigma‒Aldrich, India), sodium hydroxide, NaOH (99.99%, Sigma‒Aldrich, India) and hydrochloric acid, HCl (37%, Sigma‒Aldrich, India) were used for adjusting the pH of the sample. Deionized water was used throughout the experiment to prepare the solutions, plant extracts and wash the plants.

### Instrument and apparatus

A digital electronic balance (Model JA103P, China) with a 160 gm loading capacity was used to measure the leaf sample and all the other chemicals. A magnetic stirrer hot plate (UK) was used for stirring and maintaining the required temperature of the metal ion precursor and plant extract during the preparation of the iron nanoparticles. The extent of degradation of the dyes was monitored using a UV‒Vis spectrophotometer (CECIL CE1021, USA). After degradation, the dispersed iron nanoparticles were separated from the treated solution using an 80–2 centrifuge at a maximum speed of 5000 rpm. Fourier transform infrared spectroscopy (65 FT-IR Perkin Elmer Spectrum, USA), an ultraviolet‒visible spectrophotometer (SM-1600 spectrophotometer, USA), a powder X-ray diffractometer (Shimadzu XRD-7000S, Japan), and scanning electron microscopy (JEOL/EO-JCM-6000 plus, Japan) were used for characterization of the synthesized iron nanoparticles.

### Preparation of *Vernonia amygdalina* leaf extract

**Figure 16:**
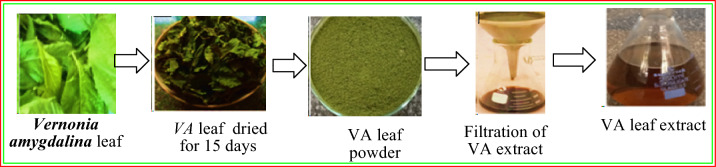
Flow diagram for the preparation of the *Vernonia amygdalina* leaf extract.

The collection of plant material for this study was initiated after obtaining the necessary permit from the EWCA Hawassa town office, with permit number EWCA-H-TT0092/23. In accordance with the World Health Organization (WHO) Quality Control Methods for Medicinal Plant Materials collection and preparation procedures^64^, mature and healthy Vernonia amygdalina leaves were collected from Hawassa Tabor Mountain Wild Life Conservation and Repair Park in the Sidama Regional State, Ethiopia, which is approximately 251 km away from Addis Ababa. The plant was authenticated by Botanist Reta Regassa, and a voucher specimen with the number TT00129/23 was deposited at the HU Herbarium, Hawassa University, for future reference. The collected leaves were cleaned thoroughly using running tap water to eliminate debris and contaminants, followed by deionized water and air drying at room temperature for one week. The aqueous extract of the plant was prepared by grinding the leaves with a mortar and pestle and boiling them in deionized water at approximately 60 °C until the color of the aqueous solution changed to brown‒red (Fig. 16). The extract was cooled to room temperature and filtered using Whatman No. 1 filter paper. Finally, the extract was stored in a refrigerator at 4 °C for further experiments.

### Biosynthesis of iron nanoparticles (FeNPs)

**Figure 17:**
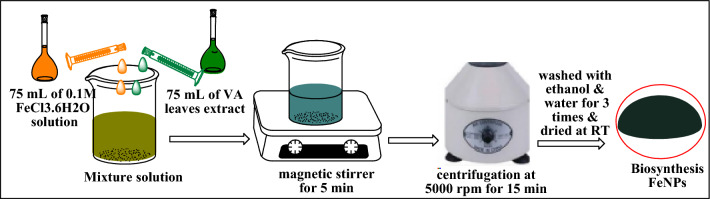
Flow diagram for the biosynthesis of FeNPs.

Iron nanoparticles were synthesized as described previously^65^ with slight modifications (as shown in Fig. 17). Ferric chloride hexahydrate (FeCl_3_·6H_2_O) was used as the metal precursor. Then, 75 mL of the *Vernonia amygdalina* leaf extract was added dropwise to 75 mL of 0.1 M FeCl_3_·6H_2_O solution at a 1:1 ratio at room temperature. The resultant mixture was stirred using a magnetic stirrer for 5 min, and the formation of a grayish black solution confirmed the synthesis of the iron nanoparticles. The nanoparticles were separated by centrifugation at 5000 rpm for 15 min and cleaned by subsequent washing with ethanol and water 3 times. The synthesized iron nanoparticles were finally dried at room temperature and stored in a sealed tight container for further use.

### Characterization of synthesized iron nanoparticles

The synthesized iron nanoparticles were characterized using Fourier transform infrared (FT-IR) spectroscopy, ultraviolet‒visible (UV‒Vis) spectroscopy, powder X-ray diffraction (XRD) and scanning electron microscopy.

### Catalytic degradation performance of iron nanoparticles

The catalytic activity of the synthesized iron nanoparticles was studied by degrading crystal violet and methylene blue in the presence of sodium borohydride as a reducing agent. To evaluate the effectiveness of the synthesized iron nanoparticles, an optimized amount of the synthesized nanoparticles was added following the addition of sodium borohydride to a beaker containing 25 mL of the prepared crystal violet and methylene blue aqueous solutions. A beaker containing a mixture of dye solution, sodium borohydride and nanoparticles was stirred on a magnetic stirrer. The mixture was subsequently centrifuged at 1500 rpm for 5 min to separate the nanoparticles from the degraded dye solution by preventing nanoparticle dispersion. The extent of degradation was monitored by measuring the absorbance before and after degradation at 590 and 664 nm for crystal violet and methylene, respectively.

### Catalyst dose

For the optimization of the catalyst dose, catalyst doses ranging from 0.010 gm to 0.125 gm with a range of 0.025 gm were used per 25 mL of 10 ppm crystal violet and methylene blue at a constant value of the other parameter. Then, the catalytic degradation efficiency was calculated.

### Initial concentration of dye

To investigate the effect of the initial concentration of dyes on the catalytic degradation efficiency, different initial concentrations of both dyes were tested (5, 10, 15, 20, 25 and 30 ppm for crystal violet and methylene blue). The catalyst dose was fixed at 0.025 gm/25 mL for crystal violet and 0.075 gm/25 mL for methylene blue. Prior to conducting these experiments, other parameters were optimized and applied while remaining constant.

### Reaction time

To investigate the total reaction time required for the complete degradation of crystal violet and methylene blue, six trials were conducted with 5 min intervals between each trial. The catalyst dose was fixed at 0.025 gm/mL for crystal violet and 0.075 gm/25 mL for methylene blue, while the initial concentrations of crystal violet and methylene blue were fixed at 20 ppm and 15 ppm, respectively. Prior to conducting these experiments, other parameters were optimized and applied.

### Concentration of sodium borohydride

The effects of the concentration of the reducing agent (NaBH_4_) on the degradation of crystal violet and methylene blue were investigated. To determine the optimum concentration of reducing agent for the best catalytic degradation, various concentrations of reducing agent were tested from 0.01 to 0.20 M. The catalyst dose was fixed at 0.025 gm/25 mL for crystal violet and 0.075 gm/25 mL for methylene blue, while the initial concentrations of crystal violet and methylene blue were fixed at 20 ppm and 15 ppm, respectively. Prior to conducting these experiments, other parameters were optimized and applied.

### pH of dyes

To study the effects of pH on the degradation of both dyes, all the other parameters were kept constant, the pH was varied from pH 2 to pH 12, and the optimum pH was investigated.

### Determination of degradation percentage

The degradation percentage of the dye solution was calculated by taking the absorbance values recorded at λ_max_ values of 590 nm and 664 nm for crystal violet and methylene blue, respectively, before and after catalytic degradation. Equation (2) is provided as follows:

2 $$Degradation\,percentage = \frac{A0 - At}{{A0}} * 100$$

where *A*_*o*_ = the initial absorbance of the dye solution before exposure to sunlight and *A*_*t*_ = the absorbance of the dye solution at time (t).

### Kinetics study

In this investigation, all the measurements were performed under optimized experimental conditions. Calibration curves were constructed for both dyes to calculate the concentrations of the dyes after degradation at different time intervals. Then, a constant concentration (initial concentration with high degradation efficiency) was used for both dyes, and the extent of degradation was studied within 5 min intervals. The concentration of dye was subsequently calculated by applying the Beer–Lambert law, as shown in Eq. (3):

3 $$A = \in bC$$

where A—Absorbance, ∈—Molar absorptivity constant, b—Path length, C—Concentration.

This equation is related to *y* = *mX* + *b*, and from the absorbance versus concentration data, the slope (m) was calculated and used to calculate the concentration after degradation. Finally, pseudozero-order, pseudo-first-order and pseudo-second-order kinetics were investigated according to Eqs. 4, 5 and 6, respectively, as mentioned below.

4 $$C{\text{o}} - {\text{C}} = k_{0} {\text{t}}$$

5 $$ln\frac{{C_{o} }}{{C_{t} }} = k_{1} t$$

6 $$\frac{1}{C} - \frac{1}{{C_{o} }} = k_{2} t$$

### Instrumental calibration

Standard stock solutions of dyes were taken for calibration of the instrument for dyes used for the experiment. For instrument calibration, first, 1000 ppm stock solution of each dye was prepared, and an intermediate standard solution containing 100 ppm was prepared in a 500 mL volumetric flask. The intermediate standards were subsequently diluted with deionized water to obtain six working standards for each dye of interest for calibration purposes.

### Study on catalyst reusability

The reusability of the catalyst was investigated during the degradation process under identical experimental conditions (at the optimum concentration of dye solution, reducing agent, catalyst dose, and time). After the completion of the reaction, the catalyst was separated by centrifugation from the reaction mixture, washed with ethanol and water, dried and reused for four consecutive cycles.

## Conclusion

Iron nanoparticles (FeNPs) were successfully synthesized by a green method using *Vernonia amygdalina* plant leaf extract as a natural reducing and capping agent. The process is relatively easy, fast, cheap, and environmentally friendly and does not require any organic solvents or other toxic reagents. Biosynthesized FeNPs were characterized with different analytical techniques, such as UV‒visible, FT-IR, XRD, and SEM, and the obtained instrumental analysis data revealed continuous absorption at the SPR in the visible range, suggesting the formation of amorphous FeNPs, the presence of various functional groups, indistinct diffraction peaks that reveal predominantly amorphous FeNPs and irregular morphology with a lack of uniform distribution in shape and size and an average particle size distribution of approximately 2.31 µm. The biosynthesized FeNPs were also applied for the catalytic degradation of MB and CV in the presence of NaBH4 and showed maximum catalytic degradation efficiencies of 94.22% and 97.47%, respectively, under optimum conditions for each dye. The kinetics study exhibited high correlation coefficients close to unity (0.999 and 0.995) for the degradation of MB and for the degradation of CV, respectively, for the zero-order pseudo kinetics model, which indicates that the model is highly suitable for the degradation of both dyes by FeNPs compared to other models. The reusability and stability of the biosynthesized nanocatalysts were studied, and the catalysts were successfully used as efficient catalysts, with a slight decrease in the degradation rate of more than four times. The results from this study showed that the use of biosynthesized FeNPs is a cost-effective, environmentally friendly, and efficient means for the highly efficient catalytic degradation of toxic pollutant dyes discharged from industries such as medicine, cosmetics, paints, plastics, and textiles.

### Supplementary Information

Supplementary Information.

## Data Availability

The data and material used to support the findings of this study are available in the supporting information (SI) and are additional available from the corresponding author upon request.

## Abbreviations

CV: Crystal violet
FeNPs: Iron nanoparticles
FTIR: Fourier transform infrared spectroscopy
pH: Power of hydrogen
SPR: Surface plasmon resonance
SEM: Scanning electron microscopy
MB: Methylene blue
Nm: Nanometer
μm: Micro meter
PSA: Particle size analysis
NPs: Nanoparticles
UV‒Vis: Ultraviolet‒visible spectroscopy
XRD: X-ray diffraction spectroscopy
EWCA: Ethiopian Wildlife Conservation Authority
LCV: Leucocrystal violet
LMB: Leucomethyene blue

## References

[1] Mirzaei H, Darroudi M (2013). Zinc oxide nanoparticles: Biological synthesis and biomedical applications. Ceram. Int..

[2] Arruda SCC, Silva ALD, Galazzi RM, Azevedo RA, Arruda MAZ (2015). Nanoparticles applied to plant science: A review. Talanta.

[3] Agarwal H, Kumar SV, Rajeshkumar S (2017). A review on green synthesis of zinc oxide nanoparticles–an eco-friendly approach. Resour. Effic. Technol..

[4] Arabi M, Ghaedi M, Ostovan A (2017). Development of a lower toxic approach based on green synthesis of water-compatible molecularly imprinted nanoparticles for the extraction of hydrochlorothiazide from human urine. ACS Sustain. Chem. Eng..

[5] Liu J, Qiao SZ, Hu QH, Lu GQ (2011). Magnetic nanocomposites with mesoporous structures: Synthesis and applications. Small.

[6] Boisseau P, Loubaton B (2011). Nanomedicine, nanotechnology in medicine. Comptes Rendus Phys..

[7] Suri SS, Fenniri H, Sing H (2007). Nanotechnology-based drug delivery systems. J. Occup. Med. Toxicol..

[8] Bhattacharya S, Saha I, Mukhopadhyay A, Chattopadhyay D, Chand U (2013). Role of nanotechnology in water treatment and purification: Potential applications and implications. Int. J. Chem. Sci. Technol..

[9] Rai M, Ingle A (2012). Role of nanotechnology in agriculture with special reference to management of insect pests. Appl. Microbiol. Biotechnol..

[10] Duncan TV (2011). Applications of nanotechnology in food packaging and food safety: Barrier materials, antimicrobials and sensors. J. Colloid Interface Sci..

[11] Stelzner T, Pietsch M, Andrä G, Falk F, Ose E, Christiansen S (2008). Silicon nanowire-based solar cells. Nanotechnology..

[12] Raj S, Jose S, Sumod U, Sabitha M (2012). Nanotechnology in cosmetics: Opportunities and challenges. J. Pharm. Bioallied Sci..

[13] Wong Y, Yuen C, Leung M, Ku S, Lam H (2006). Selected applications of nanotechnology in textiles. Autex Res. J..

[14] Ramutshatsha-Makhwedzha D, Mavhungu A, Moropeng ML, Mbaya R (2022). Activated carbon derived from waste orange and lemon peels for the adsorption of methyl orange and methylene blue dyes from wastewater. Heliyon.

[15] Wijesinghe U, Thiripuranathar G, Menaa F, Iqbal H, Razzaq A, Almukhlifi H (2021). Green synthesis, structural characterization and photocatalytic applications of ZnO nanoconjugates using *Heliotropium indicum*. Catalysts.

[16] Bhat IU, Anwar MNK, Appaturi JN (2019). Polymer based palladium nanocatalyst for the degradation of nitrate and Congo red. J. Polym. Environ..

[17] Kodoth AK, Badalamoole V (2019). Pectin based graft copolymer-ZnO hybrid nanocomposite for the adsorptive removal of crystal violet. J. Polym. Environ..

[18] Arumugham T, Kaleekkal NJ, Rana D (2018). Fabrication of novel aromatic amine functionalized nanofiltration (NF) membranes and testing its dye removal and desalting ability. Polym. Test..

[19] Khan MMR, Akter M, Amin MK, Younus M, Chakraborty N (2018). Synthesis, luminescence and thermal properties of PVA–ZnOAl_2_O_3_ composite films: Toward fabrication of sunlight-induced catalyst for organic dye removal. J. Polym. Environ..

[20] Aaga GF, Anshebo ST (2023). Green synthesis of highly efficient and stable copper oxide nanoparticles using an aqueous seed extract of Moringa stenopetala for sunlight-assisted catalytic degradation of Congo red and alizarin red s. Heliyon.

[21] Sharma P, Pant S, Rai S, Yadav RB, Dave V (2018). Green synthesis of silver nanoparticle capped with *Allium cepa* and their catalytic reduction of textile dyes: An ecofriendly approach. J. Polym. Environ..

[22] Parshetti GK, Parshetti SG, Telke AA, Kalyani DC, Doong RA, Govindwar SP (2011). Biodegradation of crystal violet by *Agrobacterium radiobacter*. J. Environ. Sci..

[23] Nezamzadeh-Ejhieh A, Amiri M (2013). CuO supported clinoptilolite toward solar photocatalytic degradation of p-aminophenol. Powder Technol..

[24] Nezamzadeh-Ejhieh A, Banan Z (2011). A comparison between the efficiency of CdS nanoparticles/zeolite A and CdO/zeolite A as catalysts in photodecolorization of crystal violet. Desalination.

[25] Kushwaha AK, Gupta N, Chattopadhyaya MC (2014). Removal of cationic methylene blue and malachite green dyes from aqueous solution by waste materials of *Daucus carota*. J. Saudi Chem. Soc..

[26] Li H, Wang G, Zhang F, Cai Y, Wang Y, Derji I (2012). Surfactant-assisted synthesis of CeO_2_ nanoparticles and their application in wastewater treatment. RSC Adv..

[27] Ghosh BK, Hazra S, Naik B, Ghosh N (2015). Preparation of Cu nanoparticle loaded SBA-15 and their excellent catalytic activity in reduction of variety of dyes. Powder Technol..

[28] Kim KH, Ihm SK (2011). Heterogeneous catalytic wet air oxidation of refractory organic pollutants in industrial wastewaters: A review. J. Hazard Mater..

[29] Sathya K, Nagarajan K, Carlin Geor Malar G, Rajalakshmi S, Raja Lakshmi P (2022). A comprehensive review on comparison among effluent treatment methods and modern methods of treatment of industrial wastewater effluent from different sources. Appl. Water Sci..

[30] Sabouri Z, Sabouri S, Tabrizi Hafez Moghaddas SS, Mostafapour A, Amiri MS, Darroudi M (2022). Facile green synthesis of Ag-doped ZnO/CaO nanocomposites with *Caccinia macranthera* seed extract and assessment of their cytotoxicity, antibacterial, and photocatalytic activity. Bioprocess Biosyst. Eng..

[31] Aroob S, Carabineiro SA, Taj MB, Bibi I, Raheel A, Javed T, Sillanpää M (2023). Green synthesis and photocatalytic dye degradation activity of CuO nanoparticles. Catalysts.

[32] Amaral PFF, Fernandes DLA, Tavares APM, Xavier ABMR, Cammarota MC, Coutinho JAP, Coelho MAZ (2004). Decolorization of dyes from textile wastewater by trametes versicolor. Environ. Technol..

[33] Mallick K, Witcomb MJ, Scurrell MS (2005). Redox catalytic property of gold nanoclusters: Evidence of an electron relay effect. Appl. Phys. A Mater..

[34] El-Sayed EM, Elkay MF, El-Latif MMA (2017). Biosynthesis and characterization of zerovalent iron nanoparticles and its application in azo dye degradation. Indian J. Chem. Technol..

[35] Khan MM, Lee J, Cho MH (2014). Au@TiO_2_ nanocomposites for the catalytic degradation of methyl orange and methylene blue: An electron relay effect. J. Ind. Eng. Chem..

[36] Wang W, Dai G, Yang H, Liu X, Chen X, Meng Z, He Q (2022). Highly efficient catalytic reduction of 4-nitrophenol and organic dyes by ultrafine palladium nanoparticles anchored on CeO_2_ nanorods. Environ. Sci. Pollut. Res..

[37] Saikia P, Miah AT, Das PP (2017). Highly efficient catalytic reductive degradation of various organic dyes by Au/CeO_2_–TiO_2_ nanohybrid. J. Chem. Sci..

[38] Abosi AO, Raseroka BH (2003). In vivo antimalarial activity of *Vernonia amygdalina*. Br. J. Biomed. Sci..

[39] Farombi EO, Owoeye O (2011). Antioxidative and chemopreventive properties of *Vernonia amygdalina* and *Garcinia biflavonoid*. Int. J. Environ. Res. Public Health.

[40] Ijeh I, Ejike C (2011). Current perspectives on the medicinal potentials of *Vernonia amygdalina* Del. J. Med. Plan Res..

[41] Liu S, Yuan L, Yue X, Zheng Z, Tang Z (2008). Recent advances in nano sensors for organophosphate pesticide detection. Adv. Powder Technol..

[42] Percival SL, Bowler PG, Russell D (2005). Bacterial resistance to silver in wound care. J. Hosp. Infect..

[43] Ashtaputrey SD, Ashtaputrey PD, Yelane N (2017). Green synthesis and characterization of copper nanoparticles derived from Murraya koenigii leaves extract. Int. J. Chem. Pharm. Sci..

[44] Sravanthi K, Ayodhya D, Yadgiri SP (2018). Green synthesis, characterization of biomaterial-supported zero-valent iron nanoparticles for contaminated water treatment. J. Anal. Sci. Technol..

[45] Roy A, Singh V, Sharma S, Ali D, Azad AK, Kumar G, Emran TB (2022). Antibacterial and dye degradation activity of green synthesized iron nanoparticles. J. Nanomater..

[46] Sravanthi K, Ayodhya D, Yadgiri Swamy P (2018). Green synthesis, characterization of biomaterial-supported zero-valent iron nanoparticles for contaminated water treatment. J. Anal. Sci. Technol..

[47] Mahnaz M, Fariedeh N, Mansor B, Ahmad R (2013). Green biosynthesis and characterization of magnetic iron oxide nanoparticles using seaweed aqueous extract. Molecules.

[48] Machado S, Pacheco J, Nouws H, Albergaria JT, Delerue-Matos C (2015). Characterization of green zero-valent iron nanoparticles produced with tree leaf extracts. Sci. Total Environ..

[49] Wang T, Lin J, Chen Z, Megharaj M, Naidu R (2014). Green synthesized iron nanoparticles by green tea and eucalyptus leaves extracts used for removal of nitrate in aqueous solution. J. Clean. Prod..

[50] Huang L, Weng X, Chen Z, Megharaj M, Naidu R (2014). Synthesis of iron-based nanoparticles using oolong tea extract for the degradation of malachite green. Spectrochim. Acta Mol..

[51] Wang Z, Fang C, Megharaj M (2014). Characterization of iron–polyphenol nanoparticles synthesized by three plant extracts and their fenton oxidation of azo dye. ACS Sustain. Chem. Eng..

[52] Önal ES, Yatkın T, Aslanov T, Ergüt M, Özer A (2019). Biosynthesis and characterization of iron nanoparticles for effective adsorption of Cr (VI). Int. J Chem. Eng..

[53] Gupta N, Singh HP, Sharma RK (2011). Metal nanoparticles with high catalytic activity in degradation of methyl orange: Electrons relay effect. J. Mol. Catal..

[54] Ji Z, Shen X, Xu Y, Zhu G, Chen K (2014). Anchoring noble metal nanoparticles on CeO_2_ modified reduced graphene oxide nanosheets and their enhanced catalytic properties. J. Colloid Interface Sci..

[55] Sabouri Z, Sabouri M, Moghaddas SSTH, Darroudi M (2023). Design and preparation of amino-functionalized core-shell magnetic nanoparticles for photocatalytic application and investigation of cytotoxicity effects. J. Environ. Health Sci. Eng..

[56] Chatterjee S, Lim S-R, Woo SH (2010). Removal of Reactive Black 5 by zero-valent iron modified with various surfactants. Chem. Eng. J..

[57] Náfrádi M, Veréb G, Firak DS, Alapi T, Garg S, Chandra A (2022). Photocatalysis: Introduction, mechanism, and effective parameters. Green Photocatalytic Semiconductors: Recent Advances and Applications.

[58] Fujioka N (2016). Linkage of iron elution and dissolved oxygen consumption with removal of organic pollutants by nanoscale zerovalent iron: Effects of pH on iron dissolution and formation of iron oxide/hydroxide layer. Chemosphere.

[59] Tran HD, Nguyen DQ, Do PT, Tran UNP (2023). Kinetics of photocatalytic degradation of organic compounds: A mini-review and new approach. RSC Adv..

[60] Jiang Z-J, Liu C-Y, Sun L-W (2005). Catalytic properties of silver nanoparticles supported on silica spheres. J. Phys. Chem. B.

[61] Khan SR, Batool M, Jamil S, Bibi S, Abid S, Janjua MRSA (2020). Synthesis and characterization of Mg–Zn bimetallic nanoparticles: selective hydrogenation of p-nitrophenol, degradation of reactive carbon black 5 and fuel additive. J. Inorg. Organomet. Polym. Mater..

[62] Hu M, Yan X, Hu X, Feng R, Zhou M (2019). Synthesis of silver decorated silica nanoparticles with rough surfaces as adsorbent and catalyst for methylene blue removal. J. Sol-Gel Sci. Technol..

[63] Arde SM, Patil AD, Mane AH, Salokhe PR, Salunkhe RS (2020). Synthesis of quinoxaline, benzimidazole and pyrazole derivatives under the catalytic influence of biosurfactant-stabilized iron nanoparticles in water. Res. Chem. Intermed..

[64] World Health Organization. *Quality control methods for medicinal plant materials* (World Health Organization, 1998).

[65] Ramy S, Yehia AM (2020). Biosynthesis and characterization of iron nanoparticles produced by *Thymus vulgaris* L. and their antimicrobial activity. Acta Bot. Croat..

