# Supplementary material for: Highly efficient catalytic degradation of organic dyes using iron nanoparticles synthesized with Vernonia Amygdalina leaf extract
Source: Sci Rep. 2024 Mar 24;14:6997. doi: 10.1038/s41598-024-57554-5 (PMC10961328; doi:10.1038/s41598-024-57554-5)
Supplement: Supplementary file 1 — Supplementary Information. [file 41598_2024_57554_MOESM1_ESM.docx]

**SUPPLEMENTARY INFROMATION**

On Full length Article

**Highly Efficient Catalytic Degradation of Organic Dyes Using Iron Nanoparticles Synthesized with *Vernonia Amygdalina* Leaf Extract**

***^1^Yohannes Shuka, ^2^Tilahun Tumiso, ^3^Alemayhu Pawulos**

1. Department of Chemistry, Natural and Computational Sciences, Mada Walabu University, P.Box 247, Bale Robe, Ethiopia
2. Department of chemistry, Natural and Computational Sciences, Hawassa University, P.Box 05, Hawassa, Ethiopia

*Corresponding Authors; [yohannesshuka@gmail.com](mailto:yohannesshuka@gmail.com) **ORCID**; [0009-0008-0311-6646](https://orcid.org/0009-0008-0311-6646)

**Appendices 1: Effect of catalyst dose**

**Table 1S:** Degradation of crystal violet by FeNPs, degradation condition of crystal violet with volume of sample 25 mL, A_o_ 0.99, concentration 10 ppm with different catalyst dose

| Catalyst dose (gm) | Triplicate | A_t_ | Degradation efficiency (%) |
| --- | --- | --- | --- |
| 0.01 | 1 | 0.110 |  |
|  | 2 | 0.111 |  |
|  | 3 | 0.113 |  |
|  | Average | **0.1113** | 88.79± 0.100504 |
| 0.025 | 1 | 0.050 |  |
|  | 2 | 0.049 |  |
|  | 3 | 0.053 |  |
|  | Average | **0.0506** | 94.55± 0.4582 |
| 0.050 | 1 | 0.074 |  |
|  | 2 | 0.076 |  |
|  | 3 | 0.075 |  |
|  | Average | **0.075** | 92.33 ± 0.090185 |
| 0.075 | 1 | 0.083 |  |
|  | 2 | 0.081 |  |
|  | 3 | 0.080 |  |
|  | Average | **0.080** | 91.786 ± 0.1527 |
| 0.1 | 1 | 0.085 |  |
|  | 2 | 0.084 |  |
|  | 3 | 0.085 |  |
|  | Average | **0.08466** | 91.435 ± 0.17321 |

Values are triplicate mean of degradation efficiency ± standard deviation

**Table S2:** Degradation of methylene blue by FeNPs, degradation condition of Methylene blue with volume of sample 25 mL, A_o_ 1.303, concentration 10 ppm with different catalyst dose

| Catalyst dose (gm) | Triplicate | | A_t_ | | Degradation efficiency (%) | |  |
| --- | --- | --- | --- | --- | --- | --- | --- |
| 0.01 | 1 | | 0.185 | |  | |  |
|  | 2 | | 0.184 | |  | |  |
|  | 3 | | 0.183 | |  | |  |
|  | Average | | **0.1846** | | 85.8733± 0.075056 | |  |
| 0.025 | 1 | | 0.115 | |  | |  |
|  | 2 | | 0.114 | |  | |  |
|  | 3 | | 0115 | |  | |  |
|  | Average | | **0.1141** | | 91.19 ± 0.046188 | |  |
| 0.050 | 1 | | 0.100 | |  | |  |
|  | 2 | | 0.100 | |  | |  |
|  | 3 | | 0.101 | |  | |  |
|  | Average | | **0.1006** | | 92.29 ± 0.04618 | |  |
| 0.075 | 1 | | 0.073 | |  | |  |
|  | 2 | | 0.075 | |  | |  |
|  | 3 | | 0.074 | |  | |  |
|  | Average | | **0.074** | | 94.266 ± 0.046188 | |  |
| 0.1 | 1 | | 0.080 | |  | |  |
|  | 2 | | 0.081 | |  | |  |
|  | 3 | | 0.079 | |  | |  |
|  | Average | | **0.08033** | | 93.85 ± 0.075056 | |  |
| 0.125 | | 1 | | 0.085 | |  | |
|  |  | 2 | | 0.084 | |  | |
|  |  | 3 | | 0.086 | |  | |
|  |  | Average | | **0.085** | | 93.47 ± 0.08 | |

Values are triplicate mean of degradation efficiency ± standard deviation

**Appendices 2: Effect of initial concentration**

**Table S3:** Degradation of crystal violate by FeNPs, degradation condition of crystal violet with catalyst dose 0.025 gm/25 mL with different initial concentration

| Initial concentration (ppm) | Triplicate | A_t_ | Degradation efficiency (%) |
| --- | --- | --- | --- |
| 5 | 1 | 0.100 |  |
|  | 2 | 0.101 |  |
|  | 3 | 0.100 |  |
|  | Average | **0.1003** | 74.3575 ± 0.131751 |
| 10 | 1 | 0.082 |  |
|  | 2 | 0.081 |  |
|  | 3 | 0.081 |  |
|  | Average | **0.08133** | 91.8125± 0.083016 |
| 15 | 1 | 0.070 |  |
|  | 2 | 0.071 |  |
|  | 3 | 0.072 |  |
|  | Average | **0.071** | 95.55 ± 0.5372 |
| 20 | 1 | 0.039 |  |
|  | 2 | 0.038 |  |
|  | 3 | 0.040 |  |
|  | Average | **0.039** | 97.88± 0.2918 |
| 25 | 1 | 0.101 |  |
|  | 2 | 0.102 |  |
|  | 3 | 0.104 |  |
|  | Average | **0.102** | 95.65± 0.5460 |
| 30 | 1 | 0.200 |  |
|  | 2 | 0.201 |  |
|  | 3 | 0.203 |  |
|  | Average | **0.2013** | 93.54 ± 0.043205 |

Values are triplicate mean of degradation efficiency ± standard deviation

**Table S4:** Degradation of methylene blue by FeNPs, degradation condition of methylene blue with catalyst dose 0.075 gm/25mLwith different initial concentration

| Initial concentration (ppm) | Triplicate | A_t_ | Degradation efficiency (%) |
| --- | --- | --- | --- |
| 5 | 1 | 0.120 |  |
|  | 2 | 0.121 |  |
|  | 3 | 0.122 |  |
|  | Average | **0.1213** | 79.57 ± 0.08124 |
| 10 | 1 | 0.141 |  |
|  | 2 | 0.140 |  |
|  | 3 | 0.140 |  |
|  | Average | **0.14033** | 89.225± 0.3785 |
| 15 | 1 | 0.050 |  |
|  | 2 | 0.051 |  |
|  | 3 | 0.049 |  |
|  | Average | **0.050** | 97.49 ± 0.04082 |
| 20 | 1 | 0.220 |  |
|  | 2 | 0.221 |  |
|  | 3 | 0.222 |  |
|  | Average | **0.2213** | 91.63± 0.03304 |
| 25 | 1 | 0.300 |  |
|  | 2 | 0.301 |  |
|  | 3 | 0.302 |  |
|  | Average | **0.301** | 90.87± 0.0244 |
| 30 | 1 | 0.430 |  |
|  | 2 | 0.431 |  |
|  | 3 | 0.432 |  |
|  | Average | **0.4313** | 88.94 ± 0.020616 |

Values are triplicate mean of degradation efficiency ± standard deviation

***Appendices 3: Effect of reaction time***

**Table S5:** Degradation of crystal violate by FeNPs, degradation condition of crystal violet with catalyst dose 0.025 gm/25 mL, A_o_ 0.991, initial concentration 20 ppm at different reaction time

| Reaction time (minute ) | Triplicate | A_t_ | Degradation efficiency (%) |
| --- | --- | --- | --- |
| 1 | 1 | 0.181 |  |
|  | 2 | 0.180 |  |
|  | 3 | 0.182 |  |
|  | Average | **0.181** | 90.85 ± 0.04028 |
| 3 | 1 | 0.162 |  |
|  | 2 | 0.160 |  |
|  | 3 | 0.163 |  |
|  | Average | **0.1616** | 91.82± 0.06238 |
| 6 | 1 | 0.120 |  |
|  | 2 | 0.118 |  |
|  | 3 | 0.119 |  |
|  | Average | **0.118** | 94.0375 ± 0.045 |
| 10 | 1 | 0.085 |  |
|  | 2 | 0.084 |  |
|  | 3 | 0.084 |  |
|  | Average | **0.08433** | 95.67± 0.1170 |
| 12 | 1 | 0.090 |  |
|  | 2 | 0.089 |  |
|  | 3 | 0.090 |  |
|  | Average | **0.090** | 95.53± 0.1436 |
| 15 | 1 | 0.092 |  |
|  | 2 | 0.093 |  |
|  | 3 | 0.092 |  |
|  | Average | **0.451** | 95.33 ± 0.02362 |
| 20 | 1 | 0.095 |  |
|  | 2 | 0.096 |  |
|  | 3 | 0.097 |  |
|  | Average | **0.09433** | 95.17 ± 0.057155 |

Values are triplicate mean of degradation efficiency ± standard deviation

**Table S6:** Degradation of methylene blue by FeNPs, degradation condition of methylene blue with catalyst dose 0.075 gm/25 mL, A_o_ 1.993, initial concentration 15 ppm at different reaction t

| Reaction time (minute ) | Triplicate | A_t_ | Degradation efficiency (%) |
| --- | --- | --- | --- |
| 5 | 1 | 0.276 |  |
|  | 2 | 0.276 |  |
|  | 3 | 0.277 |  |
|  | Average | **0.276** | 86.13 ± 0.025 |
| 10 | 1 | 0.217 |  |
|  | 2 | 0.216 |  |
|  | 3 | 0.219 |  |
|  | Average | **0.217** | 89.095± 0.062915 |
| 15 | 1 | 0.154 |  |
|  | 2 | 0.155 |  |
|  | 3 | 0.156 |  |
|  | Average | **0.155** | 92.22 ± 0.047871 |
| 20 | 1 | 0.110 |  |
|  | 2 | 0.110 |  |
|  | 3 | 0.112 |  |
|  | Average | **0.11066** | 94.4475 ± 0.04717 |
| 25 | 1 | 0.120 |  |
|  | 2 | 0.121 |  |
|  | 3 | 0.122 |  |
|  | Average | **0.121** | 93.8175± 0.04113 |
| 30 | 1 | 0.123 |  |
|  | 2 | 0.122 |  |
|  | 3 | 0.124 |  |
|  | Average | **0.1236** | 93.8175 ± 0.04113 |

Values are triplicate mean of degradation efficiency ± standard deviation

**Appendices 4: Effect of pH**

**Table S7:** Effect of pH for degradation of crystal violate by FeNPs, degradation condition of Crystal violet with catalyst dose 0.025 gm/25mL, A_o_ 1.98, initial concentration 20 ppm,10 minute reaction time at different pH

| pH | Triplicate | A_t_ | Degradation efficiency (%) |
| --- | --- | --- | --- |
| 2 | 1 | 0.250 |  |
|  | 2 | 0.249 |  |
|  | 3 | 0.253 |  |
|  | Average | **0.25067** | 87.33 ± 0.085 |
| 3 | 1 | 0.170 |  |
|  | 2 | 0.170 |  |
|  | 3 | 0.172 |  |
|  | Average | **0.1706** | 91.37± 0.04717 |
| 4 | 1 | 0.050 |  |
|  | 2 | 0.050 |  |
|  | 3 | 0.048 |  |
|  | Average | **0.050** | 97.47± 0.057735 |
| 5 | 1 | 0.078 |  |
|  | 2 | 0.079 |  |
|  | 3 | 0.077 |  |
|  | Average | **0.0780** | 96.06 ± 0.040825 |
| 6 | 1 | 0.099 |  |
|  | 2 | 0.098 |  |
|  | 3 | 0.104 |  |
|  | Average | **0.09933** | 94.98± 0.066835 |
| 7 | 1 | 0.120 |  |
|  | 2 | 0.121 |  |
|  | 3 | 0.122 |  |
|  | Average | **0.121** | 93.88 ± 0.040825 |
| 8 | 1 | 0.160 |  |
|  | 2 | 0.160 |  |
|  | 3 | 0.162 |  |
|  | Average | **0.161** | 91.8725 ±0.047871 |
| 9 | 1 | 0.190 |  |
|  | 2 | 0.190 |  |
|  | 3 | 0.191 |  |
|  | Average | **0.19033** | 90.3825±0.023629 |
| 10 | 1 | 0.210 |  |
|  | 2 | 0.211 |  |
|  | 3 | 0.212 |  |
|  | Average | **0.2113** | 89.335±0.047871 |
| 11 | 1 | 0.250 |  |
|  | 2 | 0.251 |  |
|  | 3 | 0.250 |  |
|  | Average | **0.252** | 87.3325±0.047871 |
| 12 | 1 | 0.290 |  |
|  | 2 | 0.292 |  |
|  | 3 | 0.291 |  |
|  | Average | **0.2916** | 85.2875±0.047871 |

Values are triplicate mean of degradation efficiency ± standard deviation

**Table S8:** Effect of pH for degradation of methylene blue by FeNPs, degradation condition of methylene blue with catalyst dose 0.075 gm/25 mL, A_o_ 2.646, initial concentration 20 ppm, 20 minute reaction time at different pH.

| Ph | Triplicate | A_t_ | Degradation efficiency (%) |
| --- | --- | --- | --- |
| 2 | 1 | 0.180 |  |
|  | 2 | 0.181 |  |
|  | 3 | 0.182 |  |
|  | Average | **0.181** | 90.91 ± 0.04082 |
| 3 | 1 | 0.160 |  |
|  | 2 | 0.161 |  |
|  | 3 | 0.162 |  |
|  | Average | **0.161** | 91.92± 0.040825 |
| 4 | 1 | 0.138 |  |
|  | 2 | 0.138 |  |
|  | 3 | 0.140 |  |
|  | Average | **0.1388** | 93.035 ± 0.040725 |
| 5 | 1 | 0.115 |  |
|  | 2 | 0.114 |  |
|  | 3 | 0.116 |  |
|  | Average | **0.116** | 94.22 ± 0.040825 |
| 6 | 1 | 0.130 |  |
|  | 2 | 0.131 |  |
|  | 3 | 0.132 |  |
|  | Average | **0.13066** | 93.425± 0.04203 |
| 7 | 1 | 0.145 |  |
|  | 2 | 0.146 |  |
|  | 3 | 0.147 |  |
|  | Average | **0.146** | 92.67 ± 0.040825 |
| 8 | 1 | 0.155 |  |
|  | 2 | 0.154 |  |
|  | 3 | 0.156 |  |
|  | Average | **0.155** | 92.22 ±0.04825 |
| 9 | 1 | 0.160 |  |
|  | 2 | 0.161 |  |
|  | 3 | 0.163 |  |
|  | Average | **0.1613** | 91.8325±0.1181 |
| 10 | 1 | 0.170 |  |
|  | 2 | 0.172 |  |
|  | 3 | 0.171 |  |
|  | Average | 0.171 | 91.41±0.047871 |
| 11 | 1 | 0.200 |  |
|  | 2 | 0.201 |  |
|  | 3 | 0.202 |  |
|  | Average | **0.201** | 89.91±0.0425 |
| 12 | 1 | 0.235 |  |
|  | 2 | 0.234 |  |
|  | 3 | 0.237 |  |
|  | Average | **0.235** | 88.18±0.0629 |

Values are triplicate mean of degradation efficiency ± standard deviation

**Appendix 5: Effect of sodium borohydride concentration**

**Table S9:** Degradation of crystal violet by FeNPs, degradation condition of crystal violet with catalyst dose 0.025 gm/25 mL, A_o_ 1.98, initial concentration 20 ppm at different concentration of sodium borohydride.

| Concentration of NaBH_4_ | Triplicate | At | Degradation efficiency % |
| --- | --- | --- | --- |
| 0.01 | 1 | 0.100 |  |
|  | 2 | 0.101 |  |
|  | 3 | 0.102 |  |
|  | Average | **0.101** | 94.89±0.05 |
| 0.04 | 1 | 0.080 |  |
|  | 2 | 0.081 |  |
|  | 3 | 0.083 |  |
|  | Average | **0.08133** | 95.83±0.076376 |
| 0.08 | 1 | 0.070 |  |
|  | 2 | 0.071 |  |
|  | 3 | 0.072 |  |
|  | Average | **0.071** | 96.41±0.05 |
| 0.12 | 1 | 0.045 |  |
|  | 2 | 0.047 |  |
|  | 3 | 0.046 |  |
|  | Average | **0.046** | 97.72±0.0288 |
| 0.16 | 1 | 0.055 |  |
|  | 2 | 0.056 |  |
|  | 3 | 0.057 |  |
|  | Average | **0.5566** | 97.12 ±0.05 |
| 0.2 | 1 | 0.065 |  |
|  | 2 | 0.066 |  |
|  | 3 | 0.068 |  |
|  | Average | **0.066** | 96.64 0.07637 |

Values are triplicate mean of degradation efficiency ± standard deviation

**Table S10:** Degradation of methylene blue by FeNPs, degradation condition of methylene blue with catalyst dose 0.075 gm/25 mL A_o_ 1.993, initial concentration 15 ppm at different sodium borohydride concentration

| Concentration of NaBH_4_ | Triplicate | At | Degradation efficiency % |
| --- | --- | --- | --- |
| 0.01 | 1 | 0.140 |  |
|  | 2 | 0.142 |  |
|  | 3 | 0.142 |  |
|  | Average | **0.14133** | 92.44 ±0.0366 |
| 0.04 | 1 | 0.120 |  |
|  | 2 | 0.120 |  |
|  | 3 | 0.122 |  |
|  | Average | **0.12066** | 93.92±0..051316 |
| 0.08 | 1 | 0.100 |  |
|  | 2 | 0.101 |  |
|  | 3 | 0.103 |  |
|  | Average | **0.1013** | 94.89±0.052915 |
| 0.12 | 1 | 0.057 |  |
|  | 2 | 0.058 |  |
|  | 3 | 0.059 |  |
|  | Average | **0.058** | 97.06±0.028868 |
| 0.16 | 1 | 0.081 |  |
|  | 2 | 0.083 |  |
|  | 3 | 0.082 |  |
|  | Average | **0.082** | 95.79± 0.1443 |
| 0.2 | 1 | 0.110 |  |
|  | 2 | 0.110 |  |
|  | 3 | 0.113 |  |
|  | Average | **0.111** | 94.41±0.07636 |

Values are triplicate mean of degradation efficiency ± standard deviation

**Appendix 6: Catalyst reusability**

**Table S11:** Reusability of FeNPs for the degradation of crystal violet at optimum condition

| Number of cycle | Triplicate | At | Degradation efficiency % |
| --- | --- | --- | --- |
| First | 1 | 0.080 |  |
|  | 2 | 0.081 |  |
|  | 3 | 0.082 |  |
|  | Average | **0.081** | 95.83±0.028868 |
| Second | 1 | 0.100 |  |
|  | 2 | 0.102 |  |
|  | 3 | 0.103 |  |
|  | Average | **0.1016** | 94.84±0.047258 |
| Third | 1 | 0.130 |  |
|  | 2 | 0.131 |  |
|  | 3 | 0.132 |  |
|  | Average | 0.131 | 93.62±0.26839 |
| Fourth | 1 | 0.160 |  |
|  | 2 | 0.163 |  |
|  | 3 | 0.162 |  |
|  | Average | **0.1616** | 91.84±0.026458 |

**Table S12:** Reusability of FeNPs for the degradation of methylene blue at optimum condition

| Number of cycle | Triplicate | At | Degradation efficiency % |
| --- | --- | --- | --- |
| First | 1 | 0.101 |  |
|  | 2 | 0.102 |  |
|  | 3 | 0.103 |  |
|  | Average | **0.102** | 94.46±0.028868 |
| Second | 1 | 0.130 |  |
|  | 2 | 0.131 |  |
|  | 3 | 0.132 |  |
|  | Average | **0.131** | 93.46±0.28868 |
| Third | 1 | 0.150 |  |
|  | 2 | 0.151 |  |
|  | 3 | 0.153 |  |
|  | Average | **0.1513** | 92.35±0.051962 |
| Fourth | 1 | 0.190 |  |
|  | 2 | 0.192 |  |
|  | 3 | 0.193 |  |
|  | Average | **0.192** | 90.34±0.0268868 |

Values are triplicate mean of degradation efficiency ± standard deviation

**Table S13: Calibration curve data**

| ppm of Crystal violet | Triplicate | Absorbance | ppm of Methylene blue | Triplicate | Absorbance |
| --- | --- | --- | --- | --- | --- |
| 5 | 1 | 0.390 | 5 | 1 | 0.592 |
|  | 2 | 0.391 |  | 2 | 0.591 |
|  | 3 | 0.391 |  | 3 | 0.590 |
|  | Average | **0.391**±0.001 |  | Average | **0.591**±0.001 |
| 10 | 1 | 0.99 | 10 | 1 | 1.304 |
|  | 2 | 0.98 |  | 2 | 1.302 |
|  | 3 | 1.00 |  | 3 | 1.303 |
|  | Average | **0.99**±0.01 |  | Average | **1.303**±0.001 |
| 15 | 1 | 1.52 | 15 | 1 | 1.99 |
|  | 2 | 1.55 |  | 2 | 1.98 |
|  | 3 | 1.52 |  | 3 | 2.00 |
|  | Average | **1.53**±0.01 |  | Average | **1.993**±0.01527 |
| 20 | 1 | 1.99 | 20 | 1 | 2.65 |
|  | 2 | 1.97 |  | 2 | 2.65 |
|  | 3 | 2.00 |  | 3 | 2.64 |
|  | Average | **1.98**±0.01527 |  | Average | **2.6466**±0.005774 |
| 25 | 1 | 2.51 | 25 | 1 | 3.3 |
|  | 2 | 2.53 |  | 2 | 3.31 |
|  | 3 | 2.5 |  | 3 | 3.30 |
|  | Average | **2.51**±30.01527 |  | Average | **3.3033**±0.005774 |
| 30 | 1 | 3.1 | 30 | 1 | 3.9 |
|  | 2 | 3.09 |  | 2 | 3.91 |
|  | 3 | 3.1 |  | 3 | 3.92 |
|  | Average | **3.13**±0.0608 |  | Average | **3.91**±0.01 |

Values are triplicate mean of degradation efficiency ± standard deviation

**Appendix 8: Kinetic data**

**Table S14:** Data for calculation of the dyes at different reaction time A_o_ 1.98 for crystals violet and 1.993 for Methylene blue for the degradation of crystal violet and methylene blue by FeNPs

| Dyes | Concentration (ppm) | Reaction time(min) | Triplicate | Absorbance(A_t_) |
| --- | --- | --- | --- | --- |
| Crystal violet | 20 | 1 | 1 | 0.180 |
|  |  |  | 2 | 0.181 |
|  |  |  | 3 | 0.179 |
|  |  |  | Average | **0.180** ± 0.001 |
|  |  | 3 | 1 | 0.160 |
|  |  |  | 2 | 0.162 |
|  |  |  | 3 | 0.159 |
|  |  |  | Average | **0.1603**±0.001528 |
|  |  | 6 | 1 | 0.120 |
|  |  |  | 2 | 0.120 |
|  |  |  | 3 | 0.119 |
|  |  |  | Average | **0.1006**±0.00057 |
|  |  | 10 | 1 | 0.085 |
|  |  |  | 2 | 0.084 |
|  |  |  | 3 | 0.086 |
|  |  |  | Average | **0.085**±0.001 |
| Methylene blue | 15 | 5 | 1 | 0.276 |
|  |  |  | 2 | 0.274 |
|  |  |  | 3 | 0.277 |
|  |  |  | Average | **0.2756**±0.001528 |
|  |  | 10 | 1 | 0.217 |
|  |  |  | 2 | 0.216 |
|  |  |  | 3 | 0.218 |
|  |  |  | Average | **0.217**±0.001 |
|  |  | 15 | 1 | 0.155 |
|  |  |  | 2 | 0.154 |
|  |  |  | 3 | 0.157 |
|  |  |  | Average | **0.1553**±0.001 |
|  |  | 20 | 1 | 0.110 |
|  |  |  | 2 | 0.111 |
|  |  |  | 3 | 0.112 |
|  |  |  | Average | **0.111**±0.001 |

**Figure S1:** Schematic diagram for catalytic reduction reaction and Mechanism of the catalytic degradation of CV and MB by NaBH_4_ and FeNPs sample
